# Optimization of a DNA extraction protocol for improving bacterial and fungal classification based on Nanopore sequencing

**DOI:** 10.1099/acmi.0.000754.v3

**Published:** 2024-10-07

**Authors:** May Soe Thu, Vorthon Sawaswong, Prangwalai Chanchaem, Pavit Klomkliew, Barry J. Campbell, Nattiya Hirankarn, Joanne L. Fothergill, Sunchai Payungporn

**Affiliations:** 1 Joint Chulalongkorn University–University of Liverpool Doctoral Program in Biomedical Sciences and Biotechnology, Faculty of Medicine, Chulalongkorn University, Bangkok 10330, Thailand; 2 Department of Infection Biology & Microbiomes, Institute of Infection, Veterinary & Ecological Sciences, University of Liverpool, Liverpool, L69 3GE, UK; 3 Center of Excellence in Immunology and Immune-Mediated Diseases, Department of Microbiology, Faculty of Medicine, Chulalongkorn University, Bangkok 10330, Thailand; 4 Program in Bioinformatics and Computational Biology, Graduate School, Chulalongkorn University, Bangkok 10330, Thailand; 5 Center of Excellence in Systems Microbiology, Department of Biochemistry, Faculty of Medicine, Chulalongkorn University, Bangkok 10330, Thailand; 6 Department of Clinical Infection, Microbiology and Immunology, Institute of Infection, Veterinary & Ecological Sciences, University of Liverpool, Liverpool, L69 3GE, UK

**Keywords:** 16S rRNA, 18S rRNA, bacteria, DNA extraction, fungi, microbiota, sequencing

## Abstract

Ribosomal RNA gene amplicon sequencing is commonly used to evaluate microbiome profiles in health and disease and document the impact of interventional treatments. Nanopore sequencing is attractive since it can provide greater classification at the species level. However, optimized protocols to target marker genes for bacterial and fungal profiling are needed. To achieve an increased taxonomic resolution, we developed extraction and full-length amplicon PCR-based approaches using Nanopore sequencing. Three lysis conditions were applied to a mock microbial community, including known bacterial and fungal species: ZymoBIOMICS lysis buffer (ML) alone, incorporating bead-beating (MLB) or bead-beating plus MetaPolyzyme enzymatic treatment (MLBE). In profiling of bacteria in comparison to reference data, MLB had more statistically different bacterial phyla and genera than the other two conditions. In fungal profiling, MLB had a significant increase of Ascomycota and a decline of Basidiomycota, subsequently failing to detect *Malassezia* and *Cryptococcus*. Also, a principal coordinates analysis plot by the Bray–Curtis metric showed a significant difference among groups for bacterial (*P=*0.033) and fungal (*P=*0.012) profiles, highlighting the importance of understanding the biases present in pretreatment. Overall, microbial profiling and diversity analysis revealed that ML and MLBE are more similar than MLB for both bacteria and fungi; therefore, using this specific pipeline, bead-beating is not recommended for whole gene amplicon sequencing. However, ML alone was suggested as an optimal approach considering DNA yield, taxonomic classification, reagent cost and hands-on time. This could be an initial proof-of-concept study for simultaneous human bacterial and fungal microbiome studies.

## Data Summary

Data will be available as Supplementary Materials. Sequence deposition. DNA sequence data was submitted to NCBI under submission portal of Sequence Read Archieve (SRA) and registered under BioProject ID PRJNA1040473 (individual accession numbers: SAMN38260085, SAMN38260086, SAMN38260087, SAMN38260088, SAMN38260089, SAMN38260090, SAMN38260091, SAMN38260092, SAMN38260093, SAMN38260094, SAMN38260095, SAMN38260096).

## Introduction

High-throughput sequencing (HTS) technologies have undoubtedly had a major impact on genomic research, allowing the study of not-yet culturable microbial communities [1,2]. This powerful sequencing approach has provided insight into many niches, allowing unrivalled detail into microbiomes [3,7]. Utilizing these data, the increased understanding of the importance of microbiota in maintaining human health has contributed to managing healthcare issues through beneficial modifications of the microbiome such as supplementation [8,12].

Amplicon sequencing is a typical application of HTS that effectively allows the study of genetic variation from complicated nucleotide mixtures and is more cost-effective than untargeted shotgun metagenomics [13,14]. A common approach has been targeting conserved genes, such as the 16S nuclear rRNA gene, to profile complex communities [15]. The 16S rRNA gene (full-length ~1500 bp) has nine variable regions (V1–V9), useful for determining the bacterial and archaeal profile to species level within complex biological samples [16,17]. However, the workflow is highly sensitive, and biases exist at all stages, from initial specimen collection and storage conditions [18,19] through to microbial DNA extraction [20,21], DNA sequencing [22] and bioinformatics analysis [23]. Methodological biases can cause significant variances in the observed microbial profiles, resulting in considerable variation between studies [24,25]. Standardization of methodologies has therefore been recognized as a significant necessity within industry and regulatory sectors [26].

In particular, less is known about the fungal community within the human microbiome, i.e. the ‘mycobiome’. Fungi can reside within the microbiota, with fungal signatures found in different body sites, including in the buccal cavity, the respiratory, intestinal and urinary tracts, and even breast milk [27]. However, little is known about their interactions with other micro-organisms [28], and the fungal profile in the gut accounts for <1 % of the human microbiome [29]. Even so, the global burden of fungal diseases is rising in human immunodeficiency virus (HIV)-infected patients, and infections are also commonly seen in patients with cancer, especially those receiving chemotherapy, and likewise in patients undergoing immunosuppression to support solid organ and stem cell transplantation, and any individuals taking immunomodulatory therapeutics to treat autoimmune and inflammatory diseases [30,33]. Consequently, different sequencing techniques have been used to profile the mycobiome, using markers such as the internal transcribed spacer (ITS) region of the rRNA operon, small ribosomal subunit or 18S rRNA gene, and the large subunit or 28S rRNA gene [28].

Nanopore sequencing has emerged as an appealing and expanding technique for real-time in-field sequencing of environmental and biological microbial samples, illustrating the advantage of sequencing the full-length 16S rRNA gene [34]. Its chemistry allows the study of low-abundance variants and high-heterogeneity samples by amplifying and sequencing the full-length gene, potentially providing species- or strain-level resolution [35]. In addition, this becomes practical due to the low cost and portability of long-read Nanopore sequencing platforms and the development of quick protocols and analytical pipelines [36,37].

The importance of a sample processing pipeline for different sample types has previously been identified [20]. Methods to improve DNA yield include increased cell lysis, bead-beating and/or enzymatic methods evaluated within different studies [38,39]. For short-read sequencing such as Illumina chemistry, bead-beating by ceramic or glass beads is a common method for bacterial cell wall lysis, with various protocols identified to increase the recovery of microbial DNA from faecal samples [40]. Protocols with small beads (0.1 mm) have been shown to provide better recovery of bacterial DNA, while methods with bigger beads (0.5 mm) yielded higher fungal DNA recovery [20,40], demonstrating that optimal processing to cover all microbial species within samples extensively can be challenging. A combination of bead-beating and enzymatic lysis (e.g. lysozyme) is also encouraged, providing both mechanical disruption and enzymatic degradation of bacterial cell wall peptidoglycans, particularly for Gram-positive bacteria [41,42]. However, an optimized, simultaneous extraction protocol for both bacterial and fungal communities following full-length rRNA gene sequencing has yet to be assessed.

To evaluate the efficiency of different DNA extraction techniques for the microbiome studies, one practice is the utilization of whole cell standards which can help to estimate the bias in microbiome pipelines [43]. In this study, we therefore evaluated three lysis approaches on reference mock microbiota community suspension samples containing representative bacterial and fungal species by means of a commercially available DNA extraction kit. These were subjected to Oxford Nanopore sequencing to establish the best method for comprehensive human microbiome studies.

## Methods

### Experimental design

For this study, we generated a mock microbiota community suspension using two commercially available reference standards: the first was the ZymoBIOMICS faecal reference kit with TruMatrix technology (D6323; Zymo Research) – DNA content, 6 ng µl^–1^ comprising 71 % *Bacillota* (*Firmicutes*), 23 % *Bacteroidota* (*Bacteroidetes*), 1 % *Actinomycetota* (*Actinobacteria*), 1 % *Verrucomicrobiota* (*Verrucomicrobia*) and <1 % for other phyla; and the other was the ATCC MSA-2010 mycobiome whole cell mix (American Type Culture Collection) – NGS standards; specification range, 2×10^7^ cells per vial (± 1 log), comprising ten fungal species (ATCC 36031, *Fusarium keratoplasticum*; CP046435.1, *Malassezia globosa*; D12804.1, *Cryptococcus neoformans*; JAADCK010000556.1, *Trichophyton interdigitale*; M55628.1, *Penicillium chrysogenum*; M60300.1, *Aspergillus fumigatus*; NG_062025.1, *Cutaneotrichosporon dermatis*; X51831.1, *Candida glabrata*; X53497.1, *Candida albicans*; and Z75578.1, *Saccharomyces cerevisiae*), each with 10 % of the total. For DNA extraction, a ZymoBIOMICS 96 MagBead DNA Kit (D4308; Zymo Research) was utilized.

**Fig. 1.**
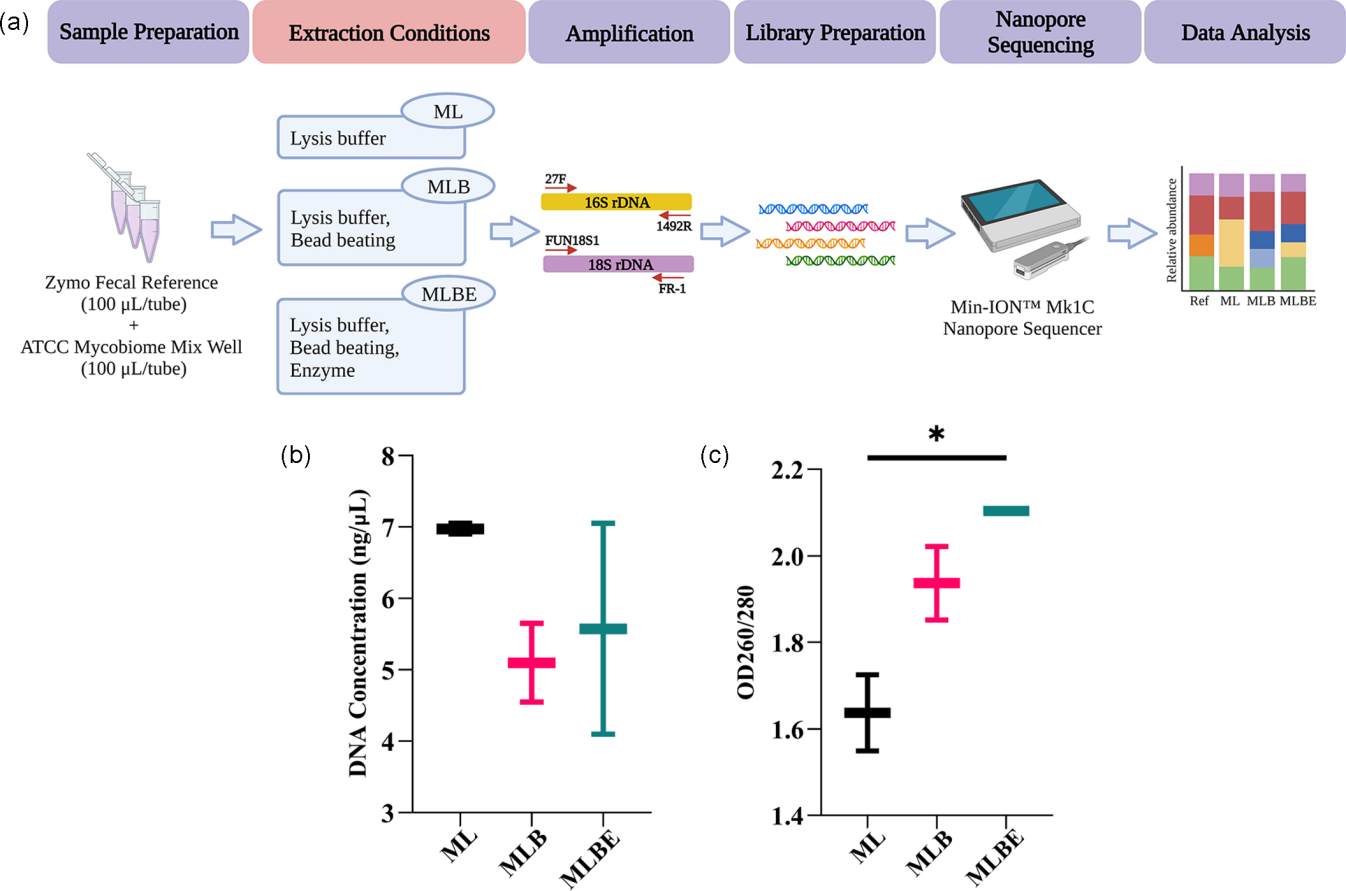
(**a**) Schematic workflow of optimizing different DNA extraction methods for microbiome study using Nanopore long-read sequencing. DNA extraction conditions were as follows: ZymoBIOMICS lysis buffer alone (ML), incorporating bead-beating (MLB) or bead-beating plus MetaPolyzyme enzymatic treatment (MLBE). Following extraction, means with error bars for (**b**) yield and (**c**) purity of isolated DNA were assessed via an Implen nanophotometer. Significant differences were observed, *P<0.05 (one-way ANOVA).

Mock microbiota community samples were prepared from equal suspension volumes of the faecal reference kit (100 µl) and the mycobiome whole cell mix (100 µl), with duplicates assigned to each of three extraction conditions: (1) use of the ZymoBIOMICS lysis buffer alone (ML); (2) lysis buffer with bead-beating (MLB); and (3) lysis buffer, bead-beating and an enzymatic treatment step using Metapolyzyme (MAC4L; Sigma-Aldrich) (MLBE). A negative control [diethyl pyrocarbonate (DEPC)-treated water] with no starting microbial reference suspension was included. Post-treatment, cellular DNA was extracted, purified, PCR amplified and then processed for sequencing (Fig. 1).

### Sample treatments and DNA extraction

All mock microbiota community suspension samples were pre-heated to 95 °C and mixed at 900 r.p.m. for 10 min using a Thermomixer (Eppendorf) as in a previous research study [44]. For ML, 750 µl ZymoBIOMICS lysis solution was added to each cell suspension sample. For MLB, suspensions were transferred into ZR Bashingbead lysis tubes containing a mix of 0.1 mm and 0.5 mm zirconium beads (S6012-50; Zymo Research) and 750 µl ZymoBIOMICS lysis solution. For the MLBE aliquots, 750 µl ZymoBIOMICS lysis solution and 5 µl Metapolyzyme were added to cell suspensions. The MLB and MLBE tubes were then subjected to bead-beating for 6 min using the TissueLyser Lt (Qiagen), with the instrument set at 50 Hz, with 1 min rest on ice after every 1.5 min of bead-beating.

All samples were centrifuged at ≥10 000 ***g*** for 1 min after treatments. For DNA purification, 200 µl of supernatant was added to 600 µl of ZymoBIOMICS MagBinding buffer and 25 µl of ZymoBIOMICS MagBinding beads, mixed well on a shaker plate at 600 r.p.m. for 10 min, then followed as per the manufacturer’s instructions. Quantity and quality of eluted DNA concentration were measured in a NanoPhotometer C40 machine (Implen), with the OD_260/280_ ratio used to determine that the sample purity was within the desired range (1.55–2.10). Samples were stored frozen for less than 1 month prior to extraction, and the DNA extracts were stored at −20 °C before amplification and sequencing.

### PCR oligonucleotide primer sequence verification using the clustalw multiple alignment tool

To evaluate the amplification performance of the primer set for the 18S rRNA gene and to determine whether we could detect all ten fungal species within the mock microbiota community, BioEdit v7.2.5 [45] was utilized. After collecting the full ribosomal sequences of the ten fungi, clustalw multiple sequence alignment (https://www.genome.jp/tools-bin/clustalw; accessed 13 June 2022) [46] was used to align with the forward and reverse primer set sequences used within this study (Fig. S1, available in the online version of this article). Multiple alignments of 18S rRNA gene sequences were conducted using the contigs from the ATCC genome assembly.

### Primer sets were optimized using different thermal cycles

PCR amplification is a key step in this approach. A primer set of the 16S rRNA gene was utilized with the Phusion plus PCR protocol as described in a previous study [47]. To ensure DNA quality, we first set up different thermal cycles, and then DNA bands with good intensity were achieved by 25 cycles, which is within the range of the manufacturer’s recommendation. A primer set of the 18S rRNA gene was selected from a previous study [48]. The same Phusion plus PCR conditions were used for gene amplification. However, DNA quality was optimized at 35 cycles for the initial amplification as in a previous research study [49].

### Amplification before Nanopore sequencing

For bacterial microbiome detection, amplification of the full-length of the 16S rRNA gene was performed using 27F and 1492R primers (Macrogen) with anchor sequences [34] (Table S1). All the amplification was duplicated for sequencing. The ZymoBIOMICS Microbial Community DNA Standard (D6305; Zymo Research) was used with the resulting plasmid as a positive control for PCR amplification, and sterile DEPC-treated water (Sigma-Aldrich) was used as template for the negative control.

For the two-step PCR, the first PCR comprised 10 ng of DNA template, 1× Phusion Plus buffer (F630XL; ThermoFisher Scientific), 200 µM dNTPs, 0.2 µM of each primer, 0.5 U PCR of Phusion Plus DNA polymerase (ThermoFisher Scientific) and DEPC water in a total volume of 20 µl. PCR was performed on an Applied Biosystems ProFlex PCR System (ThermoFisher Scientific) using the following programme: 98 °C for 30 s for initial denaturation, 25 cycles of 98 °C for 10 s, 60 °C for 10 s and 72 °C for 45 s for denaturation, annealing and extension steps respectively, and then a final extension of 72 °C for 5 min. This allowed amplicon production with overhung adapter sequences, facilitating the second round of PCR. The master mix preparation for the second PCR was as above but using only five thermal cycles. This allowed for the addition of unique indexed tags to each sample (Table S2).

For fungal mycobiome amplification, the primer set nu-SSU-0068-5′−20 (Fun18S1) and nu-SSU-1648-3′ (FR-1) (Macrogen), targeting the full-length (1.6 kb) 18S rRNA gene [48], was used (Table S1). The PCR was identical to that performed for the 16S rRNA gene but over 35 thermal cycles [50]. A non-template negative control was also included.

Amplified products were assessed for quality using 2 % (w/v) agarose gel electrophoresis in 1× Tris/borate/EDTA (TBE) buffer and purified using a QIAquick PCR purification kit (Qiagen). The purified libraries were pooled equimolarly and subsequently purified using 0.5× Agencourt AMPure XP beads (A63882; Beckman Coulter Life Sciences). Before sequencing, libraries were quantified using a Qubit 4 fluorometer and Qubit dsDNA HS (high sensitivity) assay kit (Q33239; ThermoFisher Scientific).

### Nanopore sequencing

Generated libraries underwent DNA repair, end-prep, adapter ligation and clean-up, priming and loading to the SpotON flow cell according to the manufacturer-recommended ligation sequencing amplicon protocol [SQK-LSK112; Oxford Nanopore Technologies (ONT)]. The libraries were loaded onto a MinION flow cell (R10.4; FLO-MIN112; ONT), and sequencing was performed on the MinION Mk1C nanopore sequencer (ONT). MINKNOW software v5.3.6 (ONT) was utilized for data acquisition.

### Sequence deposition

DNA sequence data were submitted to NCBI under the Sequence Read Archive (SRA) submission portal and registered under BioProject ID PRJNA1040473.

### Data processing and bioinformatic analysis

To generate the FASTQ files, the super-accuracy model of Guppy basecaller v6.0.1 (ONT) was used to basecall the FAST5 data. Read quality was evaluated using MinIONQC [51]. Then, FASTQ sequences were demultiplexed and adaptors trimmed using Porechop v0.2.4 (https://github.com/rrwick/Porechop; accessed 5 April 2022). The filtered reads were clustered, polished and taxonomically classified by NanoCLUST [52]. Bacterial taxonomy was classified using RDP database v11.5, and fungal taxonomy by an in-house curated database having ten fungal species of the mock communities (https://gofile-37314c4275.sg4.quickconnect.to/fsdownload/9mLYDFwV6/Custom18S_database; accessed 5 April 2022). Abundance taxonomic assignment data were converted into QIIME data format using the QIIME2 platform (https://qiime2.org/; accessed 5 April 2022). The normalized data files were imported into the MicrobiomeAnalyst platform [53] (www.microbiomeanalyst.ca/; accessed 5 April 2022) to assess microbial diversity between and within samples based on the relative abundance of taxa, α-diversity (Chao1 and Shannon indices) and β-diversity (Bray–Curtis index) [53].

### Subsampling

To increase the accuracy of taxonomic profile analysis and perform identical analysis on each sample, a subsampling of 10  000 read counts for the 16S rRNA gene was undertaken. Despite the low concentration at MLB1_2, all samples were taken forward for DNA sequencing to understand the impact of library quality on sequencing output. Normalization of the reads for both the 16S and 18S rRNA gene was performed by the total sum scaling method in MicrobiomeAnalyst. Rarefaction plots for both genes were created by MicrobiomeAnalyst (Fig. S4).

### Targeted amplification for detection of the genus *Malassezia*

The extracted DNA from different lysis conditions was amplified using a *Malassezia*-specific PCR primer set (MAL1F/ MAL1R; Macrogen) which provided an ~300 bp fragment containing the 5.8S rRNA gene and ITS2 (Table S1) [54,55]. PCR was performed using OnePCR Ultra Master Mix (MB208-0100; Bio-Helix), 10 pmol of each primer and 1 µl of extracted DNA in a final volume of 20 µl. PCR was performed on a ProFlex PCR System with the following programme: 94 °C for 2 min as an initial denaturation, 40 cycles of 94 °C for 20 s, 60 °C for 30 s and 72 °C for 2 min as denaturation, annealing and extension respectively, and then 72 °C for 5 min for a final extension. Sterile DEPC-treated water (Sigma-Aldrich) was also included as a negative control (Fig. S2). The purified amplicons were confirmed by Sanger sequencing on an ABI 3730XL DNA analyser (U2Bio).

### Spike-in controls

To evaluate the binding efficiency of the primer set (Fun18S1/FR-1) to *M. globosa* and other fungal species in a matrix of microbial samples, the spike-in experiment was conducted at which control samples were prepared using purified DNA isolated from spores of cultured *M. globosa* (Methods S1). We used 50, 25, 10, 5 and 0 % spike-in controls by mixing the same DNA concentration (2.35 ng µl^–1^) of combined mock controls and known *M. globosa*, followed by PCR amplification (Fig. S2) and Nanopore sequencing (Fig. S3).

### Statistical analyses

Comparison of DNA yields achieved using the three lysis methods was conducted by one-way ANOVA. To determine significant differences between groups on the abundance of bacterial and fungal profiles, a non-parametric Kruskal–Wallis test was used. Dunn’s multiple pairwise comparison test was applied to analyse differences in the abundance of each sample group to the reference, and differences were considered statistically significant at *P*<0.05. Shannon’s diversity index and the Chao1 index was used to identify α-diversity within samples. A Bray–Curtis test was used for the detection of β-diversity, followed by a permutational multivariate analysis of variance using distance matrices (PERMANOVA) test, and visualized using a principal component analysis (PCA) plot. Statistical analyses and visualization were performed using MicrobiomeAnalyst [53] (www.microbiomeanalyst.ca/; accessed 26 June 2022) and GraphPad Prism v9.0 (GraphPad Software).

## Results

### Average DNA concentration across all extraction methods was not significantly different

To ascertain the basic characteristics of the DNA following extraction, levels of DNA isolated from the mock microbial communities were determined and found to be in the range 4.1–7.05 ng µl^–1^. Average DNA concentrations with each lysis method were not different (*P*=0.4264), and DNA purity was within the desired range (OD_260/280_ 1.55–2.10; see Fig. 1b). However, intra-sample variation in DNA concentration was noted, with the second sample consistently displaying a lower concentration. Therefore, sequencing was performed by duplicating the first sample in each lysis condition.

### Concentration of DNA libraries and sequencing reads

For 16S rRNA gene amplification, the DNA concentration from two samples (ML1_2 and MLBE1_2) was around 10 ng µl^–1^, and one duplicate from each treatment (ML1_1, MLB1_1, MLBE1_1) was >20 ng µl^–1^. The range of average DNA concentration for the 18S rRNA gene amplification was 1.45–7.20 ng µl^–1^ (Table 1). One sample (MLB1_2) yielded a low DNA concentration for both the 16S rRNA gene amplification (4.98 ng µl^–1^) and the 18S rRNA gene amplification (0.638 ng µl^–1^), along with very few sequencing reads (Table 1).

**Table 1.** Quality check for DNA libraries by Qubit

	16S rRNA		18S rRNA	
Conditions	Concentration (ng µl^–1^)	Reads	Concentration (ng µl^–1^)	Reads
ML1	19.00	12 034	7.20	9755
MLB1	15.29	13 440	1.45	3727
MLBE1	19.15	14 354	7.00	7394

### Bacterial taxonomic profile using 16S rRNA gene sequencing

**Fig. 2.**
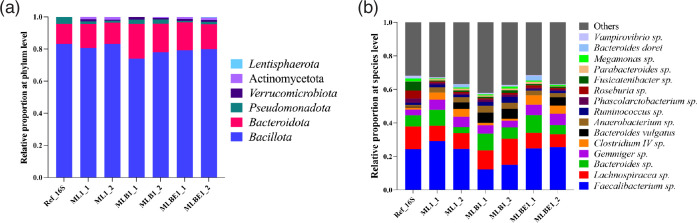
Relative bacterial abundance identified by Nanopore sequencing using DNA isolated by three different extraction methods, lysis buffer alone (ML), incorporating bead-beating (MLB) or bead-beating plus MetaPolyzyme enzymatic treatment (MLBE). Data illustrated show (**a**) all phyla and (**b**) the top 15 species identified. The initial samples, ML1, MLB1 and MLBE1, were duplicated for Nanopore sequencing, abbreviated as ML1_1, ML1_2, MLB1_1, MLB1_2, MLBE1_1 and MLBE1_2, respectively. Ref_16S, reference data of 16S gene sequencing.

At the phylum level, there were three phyla (*Bacillota*, *Bacteroidota* and *Pseudomonadota*) within the reference data, while the mock samples had not only these three phyla but also others, such as *Verrucomicrobiota* and *Actinomycetota* under all conditions, and *Lentisphaerota* only in MLBE1_2 (Fig. 2a). In total, 31 genera were identified within the reference library, and 46 genera in all samples. For data visualization, the relative abundance of identified genera was ranked, and the top 15 within the reference data (>70 % of the total identified) were presented, with all remaining identified genera (<30 %) grouped and represented as ‘Others’ (Fig. S5A). Only *Bacteroides vulgatus* and *Bacteroides dorei* were matched to the reference library at the species level, and all others were unknown species (Fig. 2b).

**Fig. 3.**
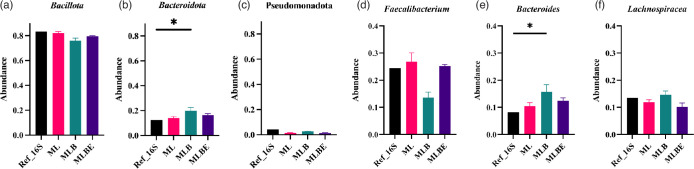
Relative abundance of the top three identified bacterial taxa identified by Nanopore sequencing of DNA isolated under different DNA extraction conditions: ZymoBIOMICS lysis buffer alone (ML), incorporating bead-beating (MLB) or bead-beating plus MetaPolyzyme enzymatic treatment (MLBE). Abundance at the phylum level: (**a**) *Bacillota*, (**b**) *Bacteroidota* and (**c**) *Pseudomonadota*; and at the species level: (**d**) *Faecalibacterium* sp.; (**e**) *Lachnospiracea* sp. and (**f**) *Bacteroides* sp. Data are expressed as the average of duplicates, and lines indicate median values. Significant differences were observed, *P<0.05 (Kruskal–Wallis test and Dunn’s multiple comparisons post-hoc test).

Looking at the individual phyla (Fig. 3a–c), no significant differences were found comparing the DNA extraction conditions ML and MLBE; however, a significant increase of *Bacteroidota* (Fig. 3b) was noted using MLB (*P=*0.0429, Table S3). We identified three common genera, *Faecalibacterium*, *Bacteroides* and *Lachnospiracea*, which comprised half of the total bacteria (Fig. S6). MLB yielded decreased levels of *Faecalibacterium* (*P*>0.05, Table S3) and an increase in *Bacteroides* (*P=*0.0412, Table S3). The abundance of *Lachnospiracea* was seen to be consistent across all extraction groups. Other significant reductions in the relative abundance of genera noted included *Phascolarctobacterium* in the ML extraction conditions (*P=*0.0412, Table S3) and *Gemmiger* using MLBE (*P=*0.0412, Table S3; Fig. S6). However, these represent a very low proportion of the reference community. No significant differences were found at the species level (Fig. S7).

**Fig. 4.**
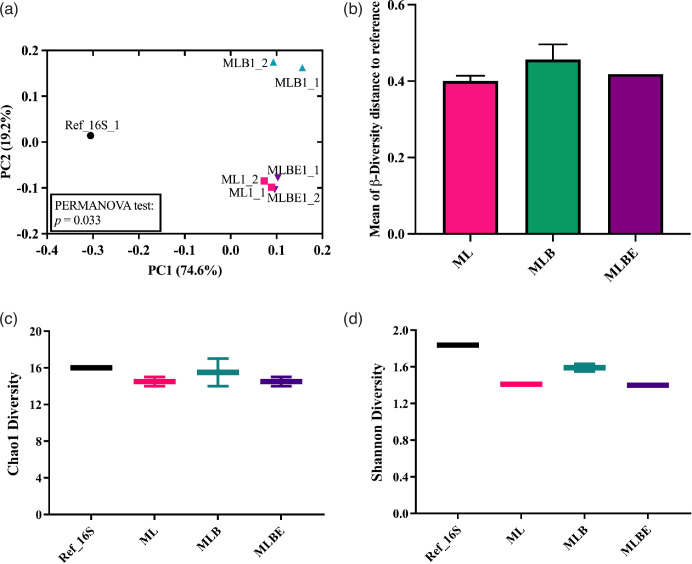
Bacterial diversity in different extraction methods: ZymoBIOMICS lysis buffer alone (ML), incorporating bead-beating (MLB) or bead-beating plus MetaPolyzyme enzymatic treatment (MLBE). Graphs illustrate (**a**) β-diversity at the species level by Bray–Curtis metrics, (**b**) the coordinate distance of the Bray–Curtis index to the reference at the bacterial species level, (**c**) α-diversity at the species level using the Chao1 index and (d) α-diversity at the species level using the Shannon index. Histogram bars represent the mean±sd. The line in the data plots indicates the median value. Significant differences were observed, *P<0.05 (Kruskal–Wallis test and Dunn’s multiple comparisons post-hoc test).

Assessment of β-diversity (*P*=0.033) revealed a significant difference between the extraction groups, with treatments ML and MLBE displaying the most similarity to the reference and each other (Fig. 4a, b). Conversely, there were no significant differences in α-diversity (Fig. 4c, d).

### Fungal taxonomic profile using 18S rRNA gene sequencing

**Fig. 5.**
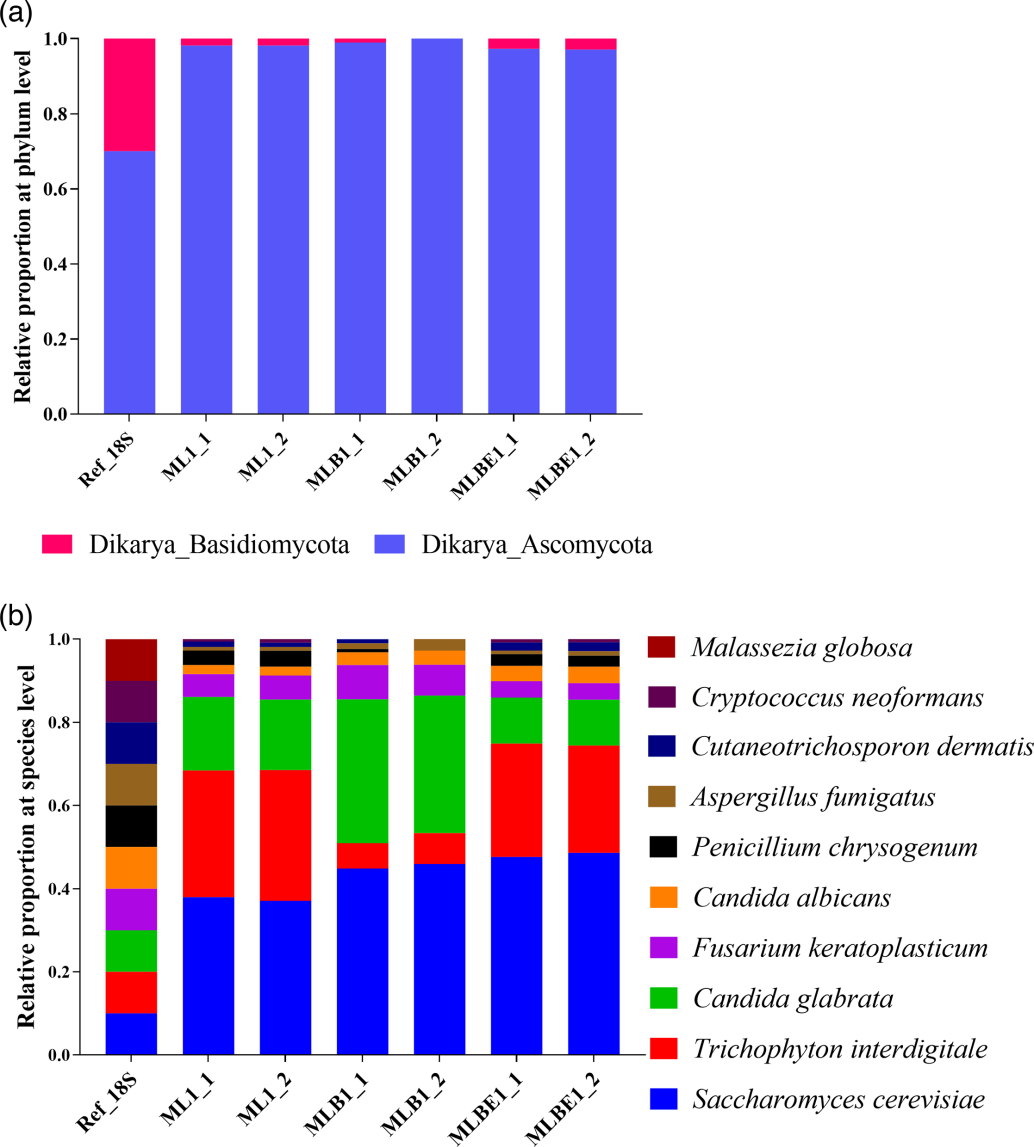
Relative bacterial abundance identified by Nanopore sequencing using DNA isolated by three different extraction methods: lysis buffer alone (ML), incorporating bead-beating (MLB) or bead-beating plus MetaPolyzyme enzymatic treatment (MLBE). Data illustrated show (**a**) all phyla and (**b**) species identified. The initial samples, ML1, MLB1 and MLBE1, were duplicated for Nanopore sequencing, abbreviated as ML1_1, ML1_2, MLB1_1, MLB1_2, MLBE1_1 and MLBE1_2, respectively. Ref_18S, reference data of 18S gene sequencing.

Using the 18S rRNA gene, the phyla Basidiomycota and Ascomycota were identified in all samples, but the relative proportions differed from the reference data (Fig. 5a). The mycobiome reference kit has ten fungal species from nine genera. However, only nine species were detected in the samples, with *M. globosa* notably absent (Fig. 5b).

**Fig. 6.**
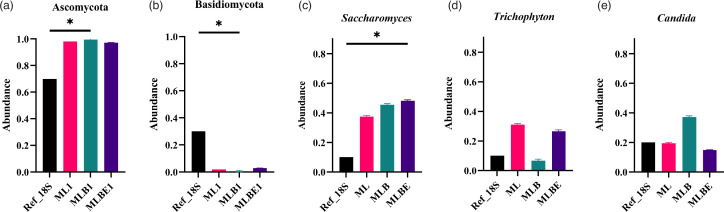
Relative abundance of top three fungal taxa identified by Nanopore sequencing of DNA isolated under different DNA extraction conditions: ZymoBIOMICS lysis buffer alone (ML), incorporating bead-beating (MLB) or bead-beating plus MetaPolyzyme enzymatic treatment (MLBE). After averaging duplicates, data illustrate the abundance of the phyla (**a**) Ascomycota and (**b**) Basidiomycota, and species (**c**) *Saccharomyces cerevisiae*, (**d**) *Trichophyton interdigitale* and (**e**) *Candida glabrata*. Lines within the data plots indicate median values. Significant differences were observed, *P<0.05 (Kruskal–Wallis test and Dunn’s multiple comparisons post-hoc test).

At an individual phylum comparison, no significant difference of Basidiomycota and Ascomycota was found with ML and MLBE; however, a significant reduction of Basidiomycota (*P=*0.0412, Table S4) and elevation of Ascomycota were observed with MLB (*P=*0.0412, Table S4) (Fig. 6a).

At the genus level, the fungal proportions varied between the reference and the samples (Fig. S5B). Comparing identified genera, MLB treatment significantly reduced *Penicillium*, *Cutaneotrichosporon* and *Cryptococcus* (*P*-value of 0.0412, 0.0412 and 0.0395, respectively, Table S4) (Fig. S8). In addition, *Saccharomyces* was highly abundant in all treatment samples, with MLBE showing a significant increase compared to the reference data (*P*=0.0412, Table S4). Furthermore, the large genus of filamentous fungi, *Fusarium*, was significantly decreased in abundance in the MLBE (*P*=0.0412, Table S4) compared to ML and MLB. At the species level (Figs 6c–e and S9), *Saccharomyces cerevisiae* and *Fusarium keratoplasticum* differed significantly between the reference and MLBE (*P=*0.0412, Table S4). Furthermore, *Candida glabrata* (*P=*0.0412, Table S4), *Penicillium chrysogenum* (*P=*0.0412, Table S4), *Cutaneotrichosporon dermatis* (*P=*0.0412, Table S4) and *Cryptococcus neoformans* (*P=*0.0395, Table S4) with MLB varied in comparison to the reference. With ML, *Candida albicans* differed compared to the reference data (*P=*0.0412, Table S4; Fig. S9).

**Fig. 7.**
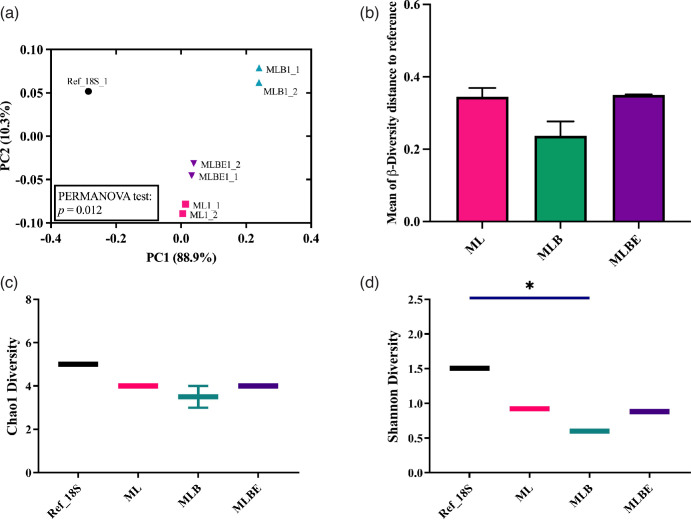
Comparison of fungal diversity following different extraction protocols: (**a**) β-diversity at the species level by Bray–Curtis metric, (**b**) coordinate distance of β-diversity to the reference at the fungal species level, (**c**) α-diversity at the species level using Chao1 index and (**d**) α-diversity at the species level using Shannon index. The bars of the histogram represent the mean with standard deviation. The line in the data plots indicates the median value. Significant differences were observed, *P<0.05 (Kruskal–Wallis test and Dunn’s multiple comparisons post-hoc test).

Fungal β-diversity was significantly different (*P*=0.012) among all lysis techniques (Fig. 7a), and the ML and MLBE methods showed the same coordinate distance to the reference (Fig. 7b). Whilst there was no difference in the Chao1 index (Fig. 7c), the Shannon diversity index showed a significant decrease in α-diversity with MLB (Fig. 7d). Overall, it revealed that ML and MLBE have more similarities to the bacterial and fungal reference data than MLB.

### The 18S rRNA primer pair can amplify all the mock community sample species

The clustalw multiple sequence alignment tool revealed perfect alignment to all the fungal species in the mock microbial samples (Fig. S1). Due to undetectability of *M. globosa* in the mock samples, the DNA extract from a known *M. globosa* colony was amplified using the same primer set (Fun18S1/FR-1, Fig. S2A), and confirmed as *M. globosa* using Nanopore sequencing (data not shown), revealing that amplification was possible using the primer set.

To ensure the feasibility of binding efficiency of the primer set in a matrix of samples, all samples having the same concentration were pooled. The pooled sample was spiked with DNA extract of known *M. globosa* at different concentrations: 50, 25, 10, 5 and 0 %. PCR was then performed using the same primer set followed by Nanopore sequencing. This revealed that the primer set could amplify the genus; however, whether the abundance was lower than 10 % could not be determined (Figs S2 and S3).

### DNA extraction provided imbalanced fungal proportions

To assess any DNA extraction bias, a specific primer set for the genus *Malassezia* (Mal1F and Mal1R) was utilized [54]. Amplified DNA bands on the gel were found in all samples along with multiple unspecific bands; therefore, a 1-in-3 dilution was used for the samples. In this experiment, ML2, MLB2 and MLBE1 samples were selected based on the band intensity to check for the genus *Malassezia* by Sanger sequencing, showing the presence of the genus in the samples under all lysis conditions. This revealed that the abundance and amplification bias in complex communities can lead to missed identification, although the fungal primer set used is able to amplify all species present.

## Discussion

Using bacterial and fungal mock samples, we compared the performance of three extraction protocols (with increasing treatment time and intensity) on the quantity and quality of DNA and microbial abundance and diversity. To assess these lysis conditions, a bacterial standard data set was obtained from Zymo Research, at which 16S rRNA gene profiling was performed on the V3–V4 region of the ribosomal gene using the Illumina MiSeq (2×300 bp) platform [56]. For the assessment of fungal communities, proportional data were provided by ATCC, which assembled their genomes using Illumina and Oxford Nanopore Hybrid Assembly. As the current study utilized Oxford Nanopore sequencing technology, it may not be directly comparable; however, these platforms have previously been shown to be comparable in detecting microbial genera [57].

The standard dataset has three bacterial phyla: *Bacillota* (83.29 %), *Bacteroidota* (12.47 %) and *Pseudomonadota* (4.24 %). In the mock microbiota community samples, these phyla accounted for over 97 % of the bacterial proportion, with the remaining 2–3 % abundance attributed to *Verrucomicrobiota* and *Actinomycetota* in all the conditions and *Lentisphaerota* only in MLBE1_2. Typically, *Bacteroidota* (73.13±22.16 %), *Bacillota* (22.2±18.66 %), *Pseudomonadota* (2.15±10.39 %), *Actinomycetota* (1.82±3 %) and *Verrucomicrobiota* (<1 %) were the top five phyla found in healthy individuals [58,59]. According to the online platform Integrated Microbial Genomes-Human Microbiome Project (IMG/HMP), *Lentisphaerota*, a member of the *Planctomycetota*–*Verrucomicrobiota*–*Chlamydiota* (PVC) superphylum, is one of the minor microbial phyla that can be identified in a healthy individual gut [60]. Since the reference kit was developed using faecal material from healthy adult donors [56], these phyla may originate from the original sample rather than a contaminant. Furthermore, in the mock samples, 46 genera and 71 species were identified while the reference data reported 31 genera and only five species. These taxonomy data suggest that higher taxonomic resolution may be achieved with entire ribosomal gene sequencing by long-read Nanopore technology compared with short-read Illumina approaches [36,37, 61]. Despite the limited information in the reference data, these techniques are comparable, as evidenced by Gehrig *et al.* [62], and therefore were used as standard bacterial data to evaluate the lysis conditions.

The ATCC mycobiome reference data had ten fungal species [63] from nine genera and two phyla (70 % relative abundance of Ascomycota and 30 % of Basidiomycota). However, in the mock samples, it was largely dominated by Ascomycota (~98 % relative abundance) and the remaining by the latter (Fig. 5a). Subsequently, the relative abundance of their downstream taxonomic levels, *Cryptococcus* and *Cutaneotrichosporon,* was very low (~1 %), and *Malassezia* was not identified under any lysis conditions (Fig. 5b). This skew may be a result of extraction bias on the 18S rRNA gene or different sequencing techniques between reference and mock samples.

In the present study, *M. globosa* was not detected through fungal profiling using amplicon sequencing of the 18S rRNA gene. However, the DNA extract from a pure colony of *M. globosa* was successfully amplified using the same primer pair (Fun18S1 and FR-1), suggesting that the primer set can amplify this species, but cannot rule out amplification bias in a complex community (Fig. S2A). Subsequently, the mock microbiota community samples were amplified using the genus-specific primer set (Mal1F and Mal1R), and the genus was identified, albeit at a very low proportion. To understand the binding efficiency of the primer set on *Malassezia* in a matrix of samples, spike-in controls with five different concentrations were prepared. We found that the primers can bind and successfully amplify *Malassezia* in a complex matrix when the DNA template contains at least 10 % of the genus (Fig. S2B and S3).

The genus *Malassezia* has been described as a difficult-to-lyse yeast [64]. Interestingly, its abundance decreased with increased bead-beating in a previous study. The relative abundance of *Malassezia* from no bead-beating to 15 min decreased from 2.8 to 0.1 %, and the genus was not present after 20 min of bead-beating [65]. This suggests an enhanced sensitivity to bead-beating, which may be further exacerbated by the need for high-quality DNA for Nanopore sequencing. The detection of *Cryptococcus* was also inconsistent in MLB. This genus is also an encapsulated yeast, and it is difficult to isolate DNA due to its thick and resistant capsule. Frau *et al.* identified that the DNA contents of *Cryptococcus neoformans* were reduced following bead-beating [44]. In a study using an Illumina whole genome sequencing approach, two cycles of rapid bead-beating (45 s, 4.5 materials s^–1^ by the RiboLyser Homogenizer) were used to extract *Cryptococcus neoformans* from the isolates [66]. Based on this, it may be assumed that the longer bead-beating time reduced the fungal species. These results show that optimizing bead-beating across different fungi is complex and optimal conditions may not be achievable for every type.

We also investigated the taxonomic differences under each lysis condition for each phylum, genus and species using the 16S and 18S rRNA gene amplicons. A significant elevation in the relative abundance of the bacterial phylum *Bacteroidota* (*P=*0.0394, Table S3) was observed in the MLB condition, while the phyla in other states were not significantly different from the reference data (Fig. 3a). At the genus level, the relative abundance of three common genera, *Bacteroides* (*P=*0.0412, Table S3)*, Clostridium* IV (*P=*0.0412, Table S3) and *Anaerobacterium* (*P=*0.0412, Table S3), was significantly elevated in MLB, and *Gemmiger* (*P=*0.0412, Table S3) in MLBE was increased significantly. *Phascolarctobacterium* (*P=*0.0412, Table S3) in ML was significantly decreased (Fig. 3b). A study by Kumar *et al.* supported the findings that the relative abundance of *Bacteroides* and *Clostridioides* was increased with a method lacking bead-beating while *Phascolarctobacterium,* an abundant genus in the gastrointesinal tract, had an elevated abundance with bead-beating [67]. In contrast, the genus *Phascolarctobacterium* was consistent across the lysis techniques (*P*>0.05, Table S3), supporting that bead-beating lysis could influence the recovery of the microbiota [67].

At the fungal phylum level (Fig. 5a), a reduction in the relative abundance of Basidiomycota and an increase in Ascomycota was observed across all the extraction and lysis conditions; subsequently, a significant decrease in the genera *Penicillium* (*P=*0.0412, Table S4), *Cutaneotrichosporon* (*P=*0.0412, Table S4) and *Cryptococcus* (*P=*0.0395, Table S4) were observed using MLB. For the MLBE condition, *Saccharomyces* (*P=*0.0412, Table S4) was increased, and *Fusarium* (*P=*0.0412, Table S4) was decreased significantly. No significant differences in these genera were observed for the ML treatment compared to the reference data. Furthermore, no significant variation was found at the species level (Fig. 5b). However, MLB showed a significant difference to the reference data (*P=*0.0412, Table S4). Considering the relative abundance of bacteria and fungi, ML and MLBE conditions yielded results most similar to the reference data. MLB treatment gave promising results for bacterial profiling but was not as promising for extracting high-quality fungal DNA.

Assessing the bacterial and fungal diversity and the PCA distance analysis (Figs4ad and 7a) revealed that ML and MLBE have more similarities to the bacterial and fungal reference data than MLB. Zhang *et al*. showed that the abundance of *Bacteroides* is higher with 1 min bead-beating and lower with more extensive beating techniques [68]. Compared to DNA isolation methods in their study, the intermittent bead-beating technique increased the proportion of *Bacteroides* and *Bacteroidota*, resulting in a more diversified bacterial population. Scharf *et al.* found that the genus *Candida* was significantly increased by 1–3 min bead-beating in fungal profiling [69] while extensive beating provided a higher abundance of fungal species but lower abundance in some species such as *Cryptococcus neoformans, Aspergillus fumigatus* and *Penicillium chrysogenum* [44]. The study revealed that certain species, such as *Saccharomyces cerevisiae* and *Candida glabrata*, displayed elevation while others showed decline. This resulted in a fungal diversity among DNA lysis approaches.

The ZymoBIOMICS Faecal Reference kit represents a bacterial quality control developed from human faecal material, and the ATCC mycobiome whole cell mixes are mock fungal communities that mimic mixed metagenomic samples [56,63]. Instead of using human samples, the current study used whole cell reference reagents to evaluate different lysis techniques. These reference community controls are quite common when analysing and benchmarking DNA extractions in microbiome studies [43]. This demonstrated variance of DNA yield under different lysis conditions and could have an impact on the outcomes of subsequent research. Limited numbers were studied in each lysis condition as this was a proof-of-concept study. Larger sample sizes, with variations in composition often seen in human samples, would yield more meaningful, and potentially more reliable results.

Bead-beating is considered critical for complete microbial lysis and accurate assessment of relative abundance and diversity, particularly short-read V3–V4 amplicons [68]. However, full-length amplicon sequencing requires better DNA quality with less tolerance for sheared DNA. This therefore leads to a balance between maximal DNA yield using methods such as bead-beating and reduced DNA quality for downstream amplification. In addition, the study evaluated existing primer sets for the full-length amplification of the 16S rRNA and 18S rRNA genes [34,48]. Using the improved Phusion plus PCR protocol for simultaneous profiling of bacteria and fungi is a strength of the work. It would be useful for fungal amplicon sequencing studies using the full-length 18S rRNA primer set (FUN18S1/FR-1).

Considering the analytical performance, reagent costs, processing time and probable biases, ML treatment was identified as a straightforward, rapid and efficient profiling method for bacterial and fungal profiles. This contrasts with many earlier studies that have highlighted the benefit of bead-beating, but we found that in the context of Nanopore sequencing, the bead-beating methodology had a negative impact on the amplification of full-length ribosomal genes compared to other methods. DNA shearing may not be problematic for short-read sequencing, but it may limit the ability to amplify full-length amplicons by Nanopore sequencing. During the experiment, negative controls were added for DNA extraction and amplification processes to consider contamination.

A limitation of the present study was that the sequencing techniques for the reference and mock samples were different, and therefore different levels of resolution were identified. There were potential technical biases during DNA extraction and sequencing processes, and all the profiling was based on a single extraction from each condition. Greater replication would yield more robust results. The results were not validated using real human faecal samples. Despite its limitations, this is an initial proof-of-concept study prior to a gut microbiome study on human faecal samples.

To summarize, the present study highlights the need to obtain high-quality DNA for profiling the human gut microbiome and mycobiome using Nanopore sequencing. This simple approach may also provide a time- and cost-efficient approach for simultaneously obtaining DNA to study bacteria and fungi residing in the gut.

## supplementary material

10.1099/acmi.0.000754.v3 Uncited Supplementary Material 1.

10.1099/acmi.0.000754.v3 Uncited Table S1.

## Abbreviations

HTS: high-throughput sequencing
ITS: internal transcribed spacer
ML: extraction with lysis buffer alone
MLB: extraction incorporating bead-beating
MLBE: extraction with bead-beating plus MetaPolyzyme enzymatic treatment

## References

[1] Ben Khedher M, Ghedira K, Rolain J-M, Ruimy R, Croce O (2022). Application and challenge of 3rd generation sequencing for clinical bacterial studies. Int J Mol Sci.

[2] Forde BM, O’Toole PW (2013). Next-generation sequencing technologies and their impact on microbial genomics. Brief Funct Genom.

[3] Yang L, Song J, Wang Y, Feng J (2021). Metagenomic next-generation sequencing for pulmonary fungal infection diagnosis: lung biopsy versus bronchoalveolar lavage fluid. Infect Drug Resist.

[4] Wu Q, Li J, Wang W, Zhou J, Wang D (2021). Next-generation sequencing reveals four novel viruses associated with Calf Diarrhea. Viruses.

[5] Wani GA, Khan MA, Dar MA, Shah MA, Reshi ZA (2021). Next generation high throughput sequencing to assess microbial communities: an application based on water quality. Bull Environ Contam Toxicol.

[6] Sahajpal NS, Mondal AK, Njau A, Petty Z, Chen J (2021). High-throughput next-generation sequencing respiratory viral panel: a diagnostic and epidemiologic tool for SARS-CoV-2 and other viruses. Viruses.

[7] Fujii T, Minami M, Watanabe T, Sato T, Kumaishi K (2021). Characterization of inter-annual changes in soil microbial flora of Panax ginseng cultivation fields in Shimane Prefecture of Western Japan by DNA metabarcoding using next-generation sequencing. J Nat Med.

[8] DAS B, Nair GB (2019). Homeostasis and dysbiosis of the gut microbiome in health and disease. J Biosci.

[9] Armour CR, Nayfach S, Pollard KS, Sharpton TJ (2019). A metagenomic meta-analysis reveals functional signatures of health and disease in the human gut microbiome. mSystems.

[10] Schmidt TSB, Raes J, Bork P (2018). The human gut microbiome: from association to modulation. Cell.

[11] Liang D, Leung R-K, Guan W, Au WW (2018). Involvement of gut microbiome in human health and disease: brief overview, knowledge gaps and research opportunities. Gut Pathog.

[12] Gilbert JA, Blaser MJ, Caporaso JG, Jansson JK, Lynch SV (2018). Current understanding of the human microbiome. Nat Med.

[13] Franzosa EA, Hsu T, Sirota-Madi A, Shafquat A, Abu-Ali G (2015). Sequencing and beyond: integrating molecular “omics” for microbial community profiling. Nat Rev Microbiol.

[14] Liu Y-X, Qin Y, Chen T, Lu M, Qian X (2021). A practical guide to amplicon and metagenomic analysis of microbiome data. Protein Cell.

[15] Ranjan R, Rani A, Metwally A, McGee HS, Perkins DL (2016). Analysis of the microbiome: advantages of whole genome shotgun versus 16S amplicon sequencing. Biochem Biophys Res Commun.

[16] Janda JM, Abbott SL (2007). 16S rRNA gene sequencing for bacterial identification in the diagnostic laboratory: pluses, perils, and pitfalls. J Clin Microbiol.

[17] Johnson JS, Spakowicz DJ, Hong B-Y, Petersen LM, Demkowicz P (2019). Evaluation of 16S rRNA gene sequencing for species and strain-level microbiome analysis. Nat Commun.

[18] Watson E-J, Giles J, Scherer BL, Blatchford P (2019). Human faecal collection methods demonstrate a bias in microbiome composition by cell wall structure. Sci Rep.

[19] Choo JM, Leong LEX, Rogers GB (2015). Sample storage conditions significantly influence faecal microbiome profiles. Sci Rep.

[20] Yang F, Sun J, Luo H, Ren H, Zhou H (2020). Assessment of fecal DNA extraction protocols for metagenomic studies. Gigascience.

[21] Lim MY, Song E-J, Kim SH, Lee J, Nam Y-D (2018). Comparison of DNA extraction methods for human gut microbial community profiling. Syst Appl Microbiol.

[22] Clooney AG, Fouhy F, Sleator RD, O’ Driscoll A, Stanton C (2016). Comparing apples and oranges?: Next generation sequencing and its impact on microbiome analysis. PLoS One.

[23] Ye SH, Siddle KJ, Park DJ, Sabeti PC (2019). Benchmarking metagenomics tools for taxonomic classification. Cell.

[24] Han D, Gao P, Li R, Tan P, Xie J (2020). Multicenter assessment of microbial community profiling using 16S rRNA gene sequencing and shotgun metagenomic sequencing. J Adv Res.

[25] Sinha R, Abu-Ali G, Vogtmann E, Fodor AA, Ren B (2017). Assessment of variation in microbial community amplicon sequencing by the Microbiome Quality Control (MBQC) project consortium. Nat Biotechnol.

[26] Stulberg E, Fravel D, Proctor LM, Murray DM, LoTempio J (2016). An assessment of US microbiome research. Nat Microbiol.

[27] Vallianou N, Kounatidis D, Christodoulatos GS, Panagopoulos F, Karampela I (2021). Mycobiome and cancer: what is the evidence?. Cancers.

[28] Nilsson RH, Anslan S, Bahram M, Wurzbacher C, Baldrian P (2019). Mycobiome diversity: high-throughput sequencing and identification of fungi. Nat Rev Microbiol.

[29] Qin J, Li R, Raes J, Arumugam M, Burgdorf KS (2010). A human gut microbial gene catalogue established by metagenomic sequencing. Nature.

[30] Sipsas NV, Kontoyiannis DP (2012). Invasive fungal infections in patients with cancer in the Intensive Care Unit. Int J Antimicrob Agents.

[31] Martínez-Martínez MU, Herrera-Van Oostdam D, Román-Acosta S, Magaña-Aquino M, Baranda-Cándido L (2012). Invasive fungal infections in patients with systemic lupus erythematosus. J Rheumatol.

[32] Silva MF, Ferriani MP, Terreri MT, Pereira RM, Magalhães CS (2015). A multicenter study of invasive fungal infections in patients with childhood-onset systemic Lupus Erythematosus. J Rheumatol.

[33] Bishu S, Su EW, Wilkerson ER, Reckley KA, Jones DM (2014). Rheumatoid arthritis patients exhibit impaired *Candida albicans*-specific Th17 responses. Arthritis Res Ther.

[34] Matsuo Y, Komiya S, Yasumizu Y, Yasuoka Y, Mizushima K (2021). Full-length 16S rRNA gene amplicon analysis of human gut microbiota using MinION. BMC Microbiol.

[35] Callahan BJ, Wong J, Heiner C, Oh S, Theriot CM (2019). High-throughput amplicon sequencing of the full-length 16S rRNA gene with single-nucleotide resolution. Nucleic Acids Res.

[36] Ciuffreda L, Rodríguez-Pérez H, Flores C (2021). Nanopore sequencing and its application to the study of microbial communities. Comput Struct Biotechnol J.

[37] Wang Y, Zhao Y, Bollas A, Wang Y, Au KF (2021). Nanopore sequencing technology, bioinformatics and applications. Nat Biotechnol.

[38] Gill C, van de Wijgert J, Blow F, Darby AC (2016). Evaluation of lysis methods for the extraction of bacterial DNA for analysis of the vaginal microbiota. PLoS One.

[39] Yuan S, Cohen DB, Ravel J, Abdo Z, Forney LJ (2012). Evaluation of methods for the extraction and purification of DNA from the human microbiome. PLoS One.

[40] Fiedorová K, Radvanský M, Němcová E, Grombiříková H, Bosák J (2019). The impact of DNA extraction methods on stool bacterial and fungal microbiota community recovery. Front Microbiol.

[41] Gryp T, Glorieux G, Joossens M, Vaneechoutte M (2020). Comparison of five assays for DNA extraction from bacterial cells in human faecal samples. J Appl Microbiol.

[42] Moss EL, Maghini DG, Bhatt AS (2020). Complete, closed bacterial genomes from microbiomes using nanopore sequencing. Nat Biotechnol.

[43] Sergaki C, Anwar S, Fritzsche M, Mate R, Francis RJ (2022). Developing whole cell standards for the microbiome field. Microbiome.

[44] Frau A, Kenny JG, Lenzi L, Campbell BJ, Ijaz UZ (2019). DNA extraction and amplicon production strategies deeply inf luence the outcome of gut mycobiome studies. Sci Rep.

[45] Hall TA (1999). Bioedit: a user-friendly biological sequence alignment editor and analysis program for windows 95/98/NT. Nucleic Acids Symp Ser.

[46] Thompson JD, Higgins DG, Gibson TJ (1994). CLUSTAL W: improving the sensitivity of progressive multiple sequence alignment through sequence weighting, position-specific gap penalties and weight matrix choice. Nucleic Acids Res.

[47] Thyagarajan S, Zhang Y, Thapa S, Allen MS, Phillips N (2020). Comparative analysis of racial differences in breast tumor microbiome. Sci Rep.

[48] Banos S, Lentendu G, Kopf A, Wubet T, Glöckner FO (2018). A comprehensive fungi-specific 18S rRNA gene sequence primer toolkit suited for diverse research issues and sequencing platforms. BMC Microbiol.

[49] Hadziavdic K, Lekang K, Lanzen A, Jonassen I, Thompson EM (2014). Characterization of the 18S rRNA gene for designing universal eukaryote specific primers. PLoS One.

[50] Romanelli AM, Fu J, Herrera ML, Wickes BL (2014). A universal DNA extraction and PCR amplification method for fungal rDNA sequence-based identification. Mycoses.

[51] Lanfear R, Schalamun M, Kainer D, Wang W, Schwessinger B (2019). MinIONQC: fast and simple quality control for MinION sequencing data. Bioinformatics.

[52] Rodríguez-Pérez H, Ciuffreda L, Flores C (2021). NanoCLUST: a species-level analysis of 16S rRNA nanopore sequencing data. Bioinformatics.

[53] Chong J, Liu P, Zhou G, Xia J (2020). Using microbiome analyst for comprehensive statistical, functional, and meta-analysis of microbiome data. Nat Protoc.

[54] Paulino LC, Tseng CH, Blaser MJ (2008). Analysis of Malassezia microbiota in healthy superficial human skin and in psoriatic lesions by multiplex real-time PCR. FEMS Yeast Res.

[55] Paulino LC, Tseng C-H, Strober BE, Blaser MJ (2006). Molecular analysis of fungal microbiota in samples from healthy human skin and psoriatic lesions. J Clin Microbiol.

[56] Zymo Research (2021). ZymoBIOMICS™ Fecal Reference with TruMatrix™ Technology. https://files.zymoresearch.com/protocols/d6323-zymobiomics_fecal_reference_protocol.pdf.

[57] Heikema AP, Horst-Kreft D, Boers SA, Jansen R, Hiltemann SD (2020). Comparison of illumina versus nanopore 16S rRNA gene sequencing of the human nasal microbiota. Genes.

[58] Li J, Si H, Du H, Guo H, Dai H (2021). Comparison of gut microbiota structure and actinobacteria abundances in healthy young adults and elderly subjects: a pilot study. BMC Microbiol.

[59] King CH, Desai H, Sylvetsky AC, LoTempio J, Ayanyan S (2019). Baseline human gut microbiota profile in healthy people and standard reporting template. PLoS One.

[60] El Kaoutari A, Armougom F, Gordon JI, Raoult D, Henrissat B (2013). The abundance and variety of carbohydrate-active enzymes in the human gut microbiota. Nat Rev Microbiol.

[61] Lu J, Zhang X, Zhang X, Wang L, Zhao R (2023). Nanopore sequencing of full rRNA operon improves resolution in mycobiome analysis and reveals high diversity in both human gut and environments. Mol Ecol.

[62] Gehrig JL, Portik DM, Driscoll MD, Jackson E, Chakraborty S (2022). Finding the right fit: evaluation of short-read and long-read sequencing approaches to maximize the utility of clinical microbiome data. Microb Genom.

[63] ATCC (2024). Mycobiome whole cell mix. https://www.atcc.org/products/msa-2010.

[64] Diaz PI, Hong B-Y, Dupuy AK, Strausbaugh LD (2017). Mining the oral mycobiome: methods, components, and meaning. Virulence.

[65] Cullen JT, Lawlor PG, Cormican P, Crispie F, Gardiner GE (2022). Optimisation of a bead-beating procedure for simultaneous extraction of bacterial and fungal DNA from pig faeces and liquid feed for 16S and ITS2 rDNA amplicon sequencing. Animal - Open Space.

[66] Rhodes J, Beale MA, Fisher MC (2014). Illuminating choices for library prep: a comparison of library preparation methods for whole genome sequencing of Cryptococcus neoformans using Illumina HiSeq. PLoS One.

[67] Kumar A, Gravdal K, Segal JP, Steed H, Brookes MJ (2022). Variability in the pre-analytical stages influences microbiome laboratory analyses. Genes.

[68] Zhang B, Brock M, Arana C, Dende C, van Oers NS (2021). Impact of bead-beating intensity on the genus- and species-level characterization of the gut microbiome using amplicon and complete 16S rRNA gene sequencing. Front Cell Infect Microbiol.

[69] Scharf S, Bartels A, Kondakci M, Pfeffer K, Henrich B (2020). Introduction of a bead beating step improves fungal DNA extraction from selected patient specimens. Int J Med Microbiol.

