# Supplementary material for: Optimization of a DNA extraction protocol for improving bacterial and fungal classification based on Nanopore sequencing
Source: Access Microbiol. 2024 Oct 7;6(10):000754.v3. doi: 10.1099/acmi.0.000754.v3 (PMC11457918; doi:10.1099/acmi.0.000754.v3)

Optimisation of DNA extraction protocol for improving the bacterial and fungal classification based on Nanopore sequencing. Thu MS, Sawaswong V, Chanchaem P, Klomkliew P, Campbell BJ, Hirankarn N, Fothergill JL, Payungporn S.

Supplementary Materials

Table S1: Primers used for PCR amplification of marker-specific genes

Primers	*Oligonucleotide primer Sequences (5´ to 3´)	Target Region	Target Gene	Reference
S-D-BACT-0008-C-S-20 (27F)	TTT CTG TTG GTG CTG ATA TTG CAG RGT TYG ATY MTG GCT CAG	V1-V9	16S rRNA	[1]
S-D-BACT-1492-A-A-22 (1492R)	ACT TGC CTG TCG CTC TAT CTT CCG GYT ACC TTG TTA CGA CTT	V1-V9	16S rRNA	[1]
NU-SSU-0068-5′-20 (FUN18S1)	TTT CTG TTG GTG CTG ATA TTG CCC ATG CAT GTC TAA GTW TAA	V1-V8	18S rRNA	[2]
NU-SSU-1648-3′ (FR-1)	ACT TGC CTG TCG CTC TAT CTT CAN CCA TTC AAT CGG TAN T	V1-V8	18S rRNA	[2]
MAL1F	TCTTTGAACGCACCTTGC	-	5.8S rRNA, ITS2	[3]
MAL1R	AHAGCAAATGACGTATCATG	-	5.8S rRNA, ITS2	[3]

*Sequences underlined indicate the primer binding site

[1] Matsuo Y, Komiya S, Yasumizu Y, Yasuoka Y, Mizushima K, Takagi T, et al. Full-length 16S rRNA gene amplicon analysis of human gut microbiota using MinION nanopore sequencing confers species-level resolution. BMC Microbiol. 2021;21(1):35.

[2] Banos S, Lentendu G, Kopf A, Wubet T, Glöckner FO, Reich M. A comprehensive fungi-specific 18S rRNA gene sequence primer toolkit suited for diverse research issues and sequencing platforms. BMC Microbiology. 2018;18(1):190.

[3] Paulino, L.C., C.H. Tseng, and M.J. Blaser, Analysis of Malassezia microbiota in healthy superficial human skin and in psoriatic lesions by multiplex real-time PCR. FEMS Yeast Res, 2008. 8(3): p. 460-71.

Table S2: Barcode adapters used for preparation of sequencing libraries

*Adapters	Oligonucleotide primer sequences (5´ to 3´)
BC36/RB36	ATGTCCCAGTTAGAGGAGGAAACA
BC37/RB37	GCTTGCGATTGATGCTTAGTATCA
BC38/RB38	ACCACAGGAGGACGATACAGAGAA
BC39/RB39	CCACAGTGTCAACTAGAGCCTCTC
BC40/RB40	TAGTTTGGATGACCAAGGATAGCC
BC41/RB41	GGAGTTCGTCCAGAGAAGTACACG
BC42/RB42	CTACGTGTAAGGCATACCTGCCAG
BC43/RB43	CTTTCGTTGTTGACTCGACGGTAG
BC44/RB44	AGTAGAAAGGGTTCCTTCCCACTC
BC45/RB45	GATCCAACAGAGATGCCTTCAGTG
BC46/RB46	GCTGTGTTCCACTTCATTCTCCTG
BC47/RB47	GTGCAACTTTCCCACAGGTAGTTC
BC48/RB48	CATCTGGAACGTGGTACACCTGTA

*The adapters were obtained from PCR Barcoding Expansion 1-96 (EXP-PBC096) kit (Oxford Nanopore Technologies, UK).

Optimisation of DNA extraction protocol for improving the bacterial and fungal classification based on Nanopore sequencing. Thu MS, Sawaswong V, Chanchaem P, Klomkliew P, Campbell BJ, Hirankarn N, Fothergill JL, Payungporn S.

Table S3: A table of p-values for the comparison of different lysis conditions at the phylum, genus and species level of 16S rDNA

p-values*	Ref vs ML	Ref vs MLB	Ref vs MLBE
**Phylum of 16S rDNA**			
Firmicutes	ns	ns	ns
Bacteroides	ns	0.0429	ns
Proteobacteria	ns	ns	ns
**Genus of 16S rDNA**			
Faecalibacterium	ns	ns	ns
Bacteroides	ns	0.0412	ns
Lachnospiracea	ns	ns	ns
Clostridium IV	ns	0.0412	ns
Gemmiger	ns	ns	0.0412
Anaerobacterium	ns	0.0412	ns
Blautia	ns	ns	ns
Alistipes	ns	ns	ns
Roseburia	ns	ns	ns
Ruminococcus	ns	ns	ns
Parabacteroides	ns	ns	ns
Phascolarctobacterium	0.0412	ns	ns
Fusicatenibacter	ns	ns	ns
**Species of 16S rDNA**			
Faecalibacterium sp.	ns	ns	ns
Lachnospiracea sp.	ns	ns	ns
Bacteroides sp.	ns	ns	ns
Gemmiger sp.	ns	ns	0.0412
Clostridium IV sp.	ns	ns	ns
Bacteroides vulgatus	ns	ns	ns
Anaerobacterium sp.	ns	0.0412	ns
Ruminococcus sp.	ns	ns	ns
Phascolarctobacterium sp.	0.0412	ns	ns
Roseburia sp.	ns	ns	ns
Fusicatenibacter sp.	ns	ns	ns
Parabacteroides sp.	ns	ns	ns
Megamonas sp.	ns	ns	ns
Bacteroides dorei	ns	ns	ns
Vampirovibrio sp.	ns	ns	ns

*The p-values were obtained from Dunn's multiple comparison test. ns, not significant ($p > 0.05$).

Optimisation of DNA extraction protocol for improving the bacterial and fungal classification based on Nanopore sequencing. Thu MS, Sawaswong V, Chanchaem P, Klomkliew P, Campbell BJ, Hirankarn N, Fothergill JL, Payungporn S.

Table S4: A table of p-values for the comparison of different lysis conditions at the phylum, genus and species level of 18S rDNA

p-values*	Ref vs ML	Ref vs MLB	Ref vs MLBE
**Phylum of 18S rDNA**			
Basidiomycota	ns	0.0412	ns
Ascomycota	ns	0.0412	ns
**Genus of 18S rDNA**			
Saccharomyces	ns	ns	0.0412
Trichophyton	ns	ns	ns
Candida	ns	ns	ns
Fusarium	ns	ns	0.0412
Penicillium	ns	0.0412	ns
Aspergillus	ns	ns	ns
Cutaneotrichosporon	ns	0.0412	ns
Cryptococcus	ns	0.0395	ns
**Species of 18S rDNA**			
Saccharomyces cerevisiae	ns	ns	0.0412
Trichophyton interdigitale	ns	ns	ns
Candida glabrata	ns	0.0412	ns
Fusarium keratoplasticum	ns	ns	0.0412
Candida albicans	0.0412	ns	ns
Penicillium chrysogenum	ns	0.0412	ns
Aspergillus fumigatus	ns	ns	ns
Cutaneotrichosporon dermatis	ns	0.0412	ns
Cryptococcus neoformans	ns	0.0395	ns

*The p-values were obtained from Dunn’s multiple comparison test. ns, not significant ($p > 0.05$).

Figure S1. **CLUSTALW Multiple sequence alignment.** Multiple alignment of the 18S rRNA gene sequences in mock microbiota community, aligned to **A)** forward and **B)** reverse oligonucleotide primers using CLUSTALW (www.genome.jp/tools-bin/clustalw ; accessed 13 June 2022). Full sequences of 7 species were found from SILVA database (www.arb-silva.de/; accessed 13 June 2022). For *Malassezia*, the full 18S rRNA was retrieved using Nucleotide Basic Local Alignment Search Tool (BLASTn). For *Trichophytum* and *Fusarium* genera, pair-alignment was performed using the contigs from ATCC genome assembly, then made a BLASTn at NCBI (https://blast.ncbi.nlm.nih.gov/Blast.cgi ; accessed on 13 June 2022).

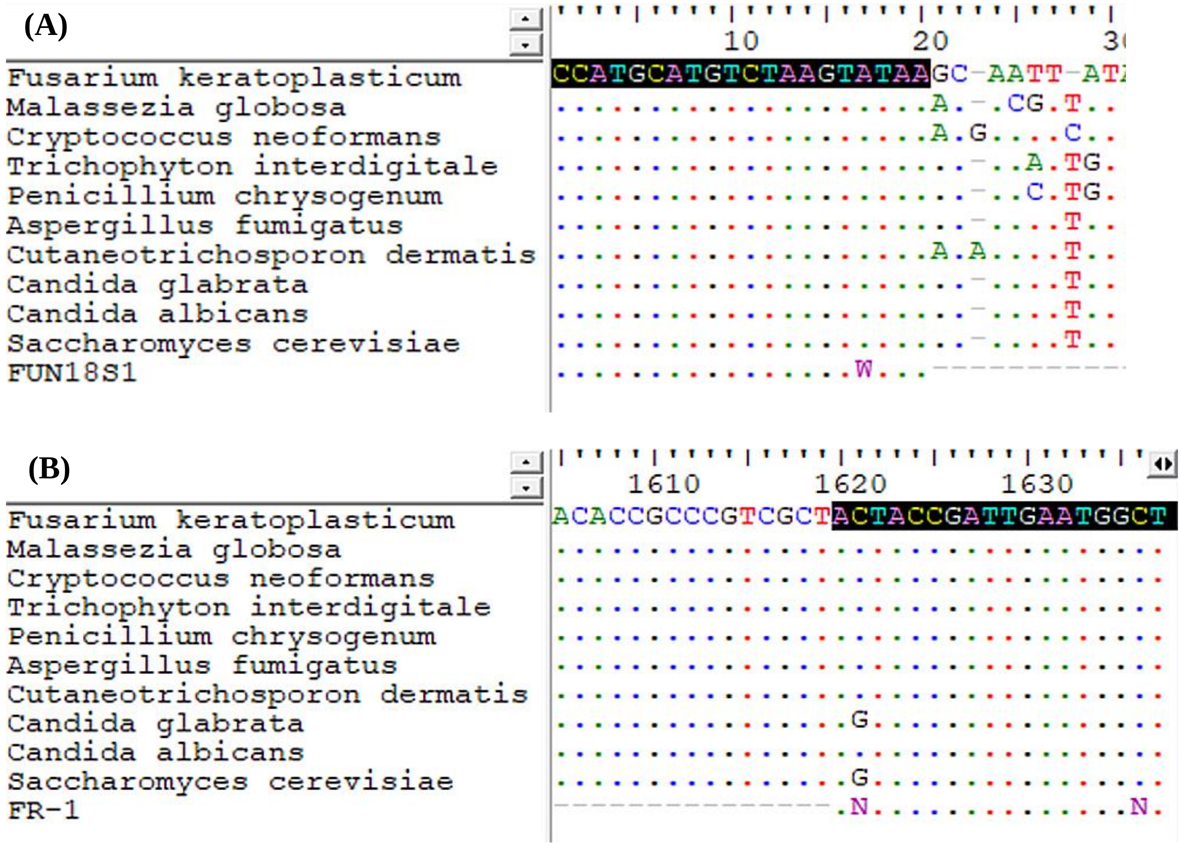

Methods S1: Culture of, and isolation of DNA from, *Malassezia globosa*. *M. globosa* was grown initially on modified Dixon's (mDixon) agar containing 26 mg/L chloramphenicol for 3–4 d, at 32 °C, as per conditions previously described for *Malassezia* sp. [Frau et al., 2019]. Briefly, mDixon broth was then inoculated with a single colony and incubated at 32 °C, shaking at 200 rpm for 48 h. Spores were harvested, and counted with a Neubauer-improved haemocytometer. And then extracted using a QIAamp Fast DNA mini kit (Qiagen; Hilden, Germany). Following extraction, DNA was quantified with a Qubit (Qubit dsDNA HS assay Kit; Life Technologies).

Frau, A., Kenny, J.G., Lenzi, L. *et al.* DNA extraction and amplicon production strategies deeply influence the outcome of gut mycobiome studies. *Sci Rep* 2019; **9**, 9328

Figure S2. PCR amplification of *Malassezia* from isolated DNA extracted under different lysis conditions. Gel electrophoresis of PCR amplicons generated using, A) the first PCR product in 2-step amplification to evaluate the amplification bias of the primer set on *Malassezia globosa* (MG) at which a negative control was performed but not shown here; B) using 18S rRNA primer set (FUN18S1/FR-1) on samples spiked with known amounts of purified *M. globosa* DNA. Different spike-in concentrations: 50%, 25%, 10%, 5%, and 0% of the mock community DNA library, were prepared using the same concentration of a mixture of mock control and known *M. globosa* samples; and C) using *Malassezia*-specific PCR primers MAL1F (5'-TCTTTGAACGCACCTTGC-3') and MAL1R (5'- AHAGCAAATGACGTATCATG-3') on all samples prior to Sanger sequencing. Positive (Pos) control, with 2.35 ng/µL DNA isolate from *M. globosa*. Negative control (Neg), with no DNA template included in the amplification.

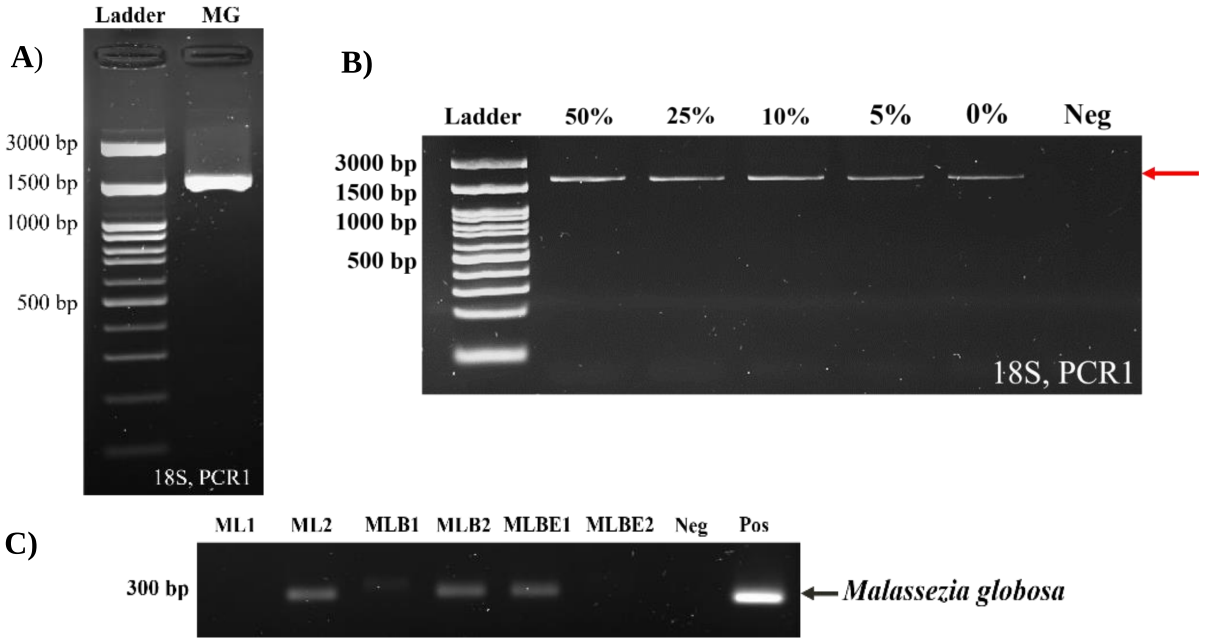

Figure S3: Comparison of fungal abundance at spike-in controls. Different spike-in concentration: 50%, 25%, 10%, 5%, and 0%, were prepared using the same concentration of a mixture of mock microbiota community and known proportions of *M. globosa* samples.

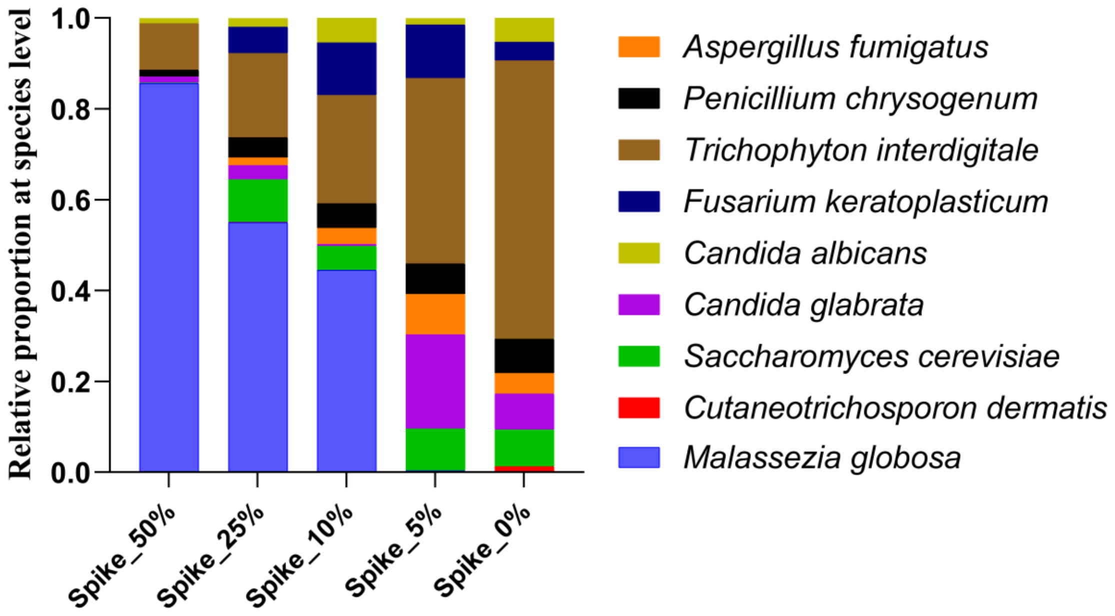

Figure S4: Rarefaction curves showing observed species richness based on (**A**) 16S rRNA, and (**B**) 18S rRNA gene sequencing, created by MicrobiomeAnalyst (www.microbiomeanalyst.ca/, accessed 04 June 2022). Three sample lysis conditions were applied to a mock microbial community including known bacterial and fungal species; using the 96 MagBead DNA lysis buffer (ML) alone, incorporating bead-beating (MLB), or bead-beating following MetaPolyzyme enzymatic pre-treatment (MLBE). The initial samples: ML1, MLB1, and MLBE1 were duplicated for Nanopore sequencing and abbreviated as ML1_1, ML1_2, MLB1_1, MLB1_2, MLBE1_1 and MLBE1_2. The 16S and 18S rRNA gene sequencing reference data were abbreviated as Ref_16S and Ref_18S, respectively. Species richness is the count of each operational taxonomy unit (OTUs) in each reference (Ref_16S and Ref_18S) and sample. The sequence sample size is the total read count of each reference (Ref_16S and Ref_18S) and sample.

(A)

(B)

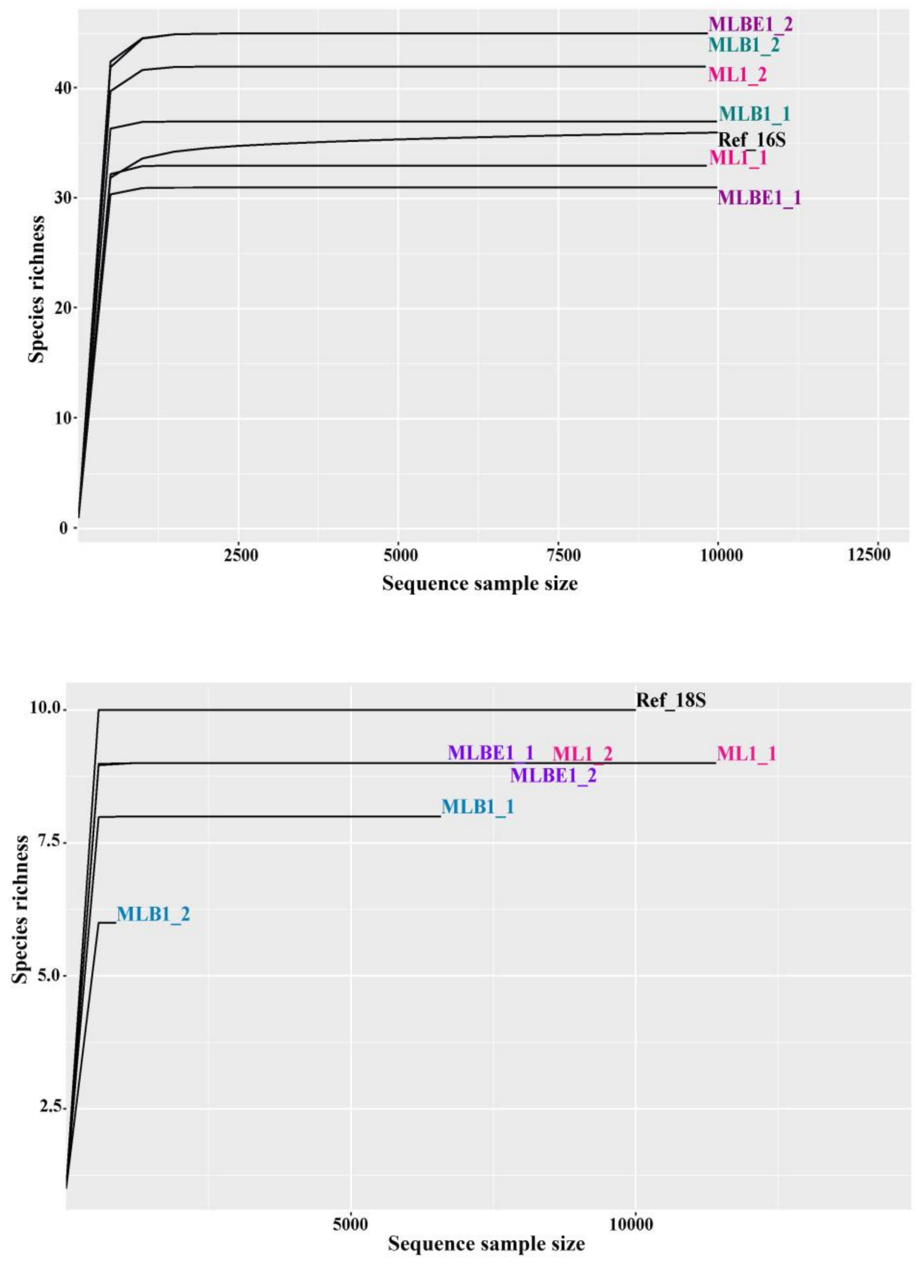

Figure S5: Relative abundance identified by Nanopore long-read sequencing using DNA isolated by 3 different extraction methods, lysis buffer alone (ML), incorporating bead-beating (MLB), or bead-beating plus MetaPolyzyme enzymatic treatment (MLBE). Data illustrated shows (A) the top 15 bacterial genera, and (B) all the fungal genera identified. The initial samples: ML1, MLB1, and MLBE1 were duplicated for Nanopore sequencing, abbreviated as ML1_1, ML1_2, MLB1_1, MLB1_2, MLBE1_1 and MLBE1_2, respectively. The reference data of 16S and 18S rRNA gene sequencing was abbreviated as Ref_16S and Ref_18S, respectively.

(A)

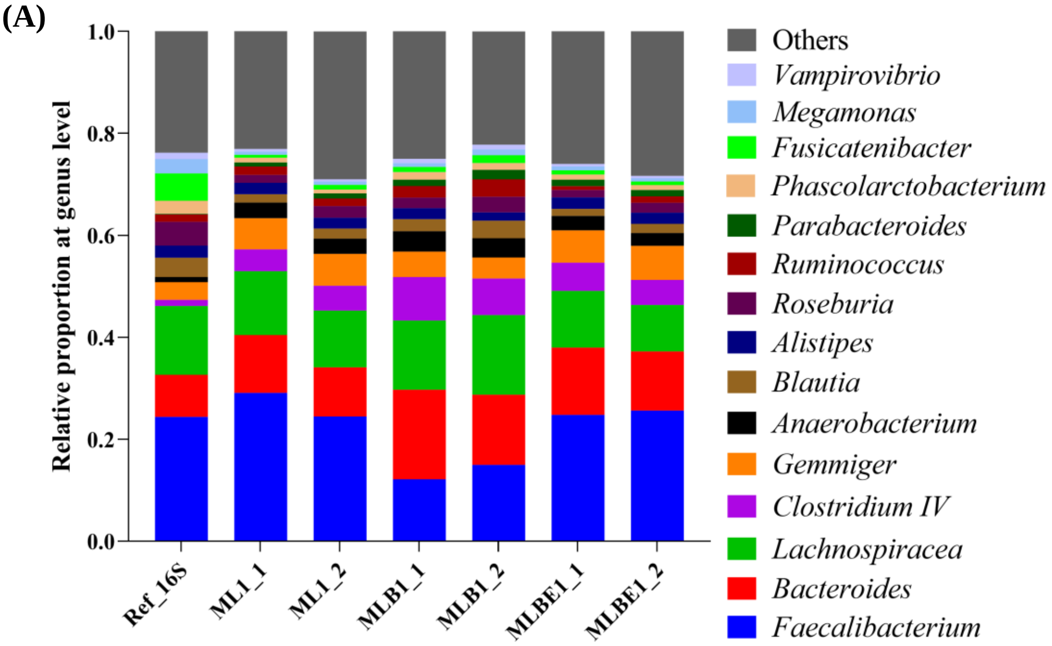

(B)

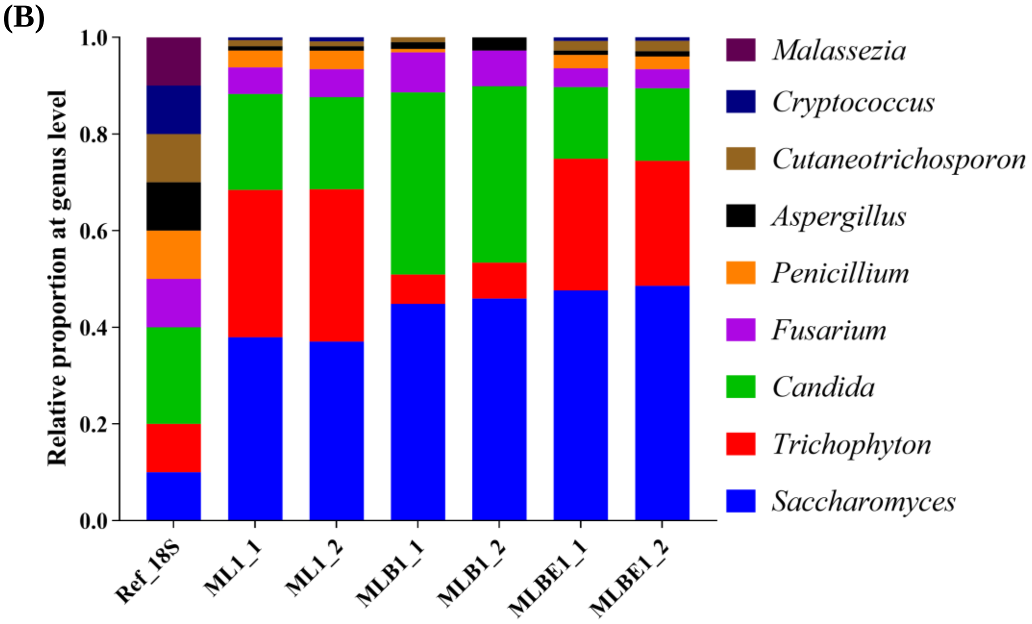

Figure S6: Relative abundance of Top 15 bacterial taxonomy at the genus level. Kruskal-Wallis statistic test; asterisks indicate adjusted *p*-value < 0.05 in Dunn's multiple comparisons posthoc test. Error bars are indicated on top of the boxes.

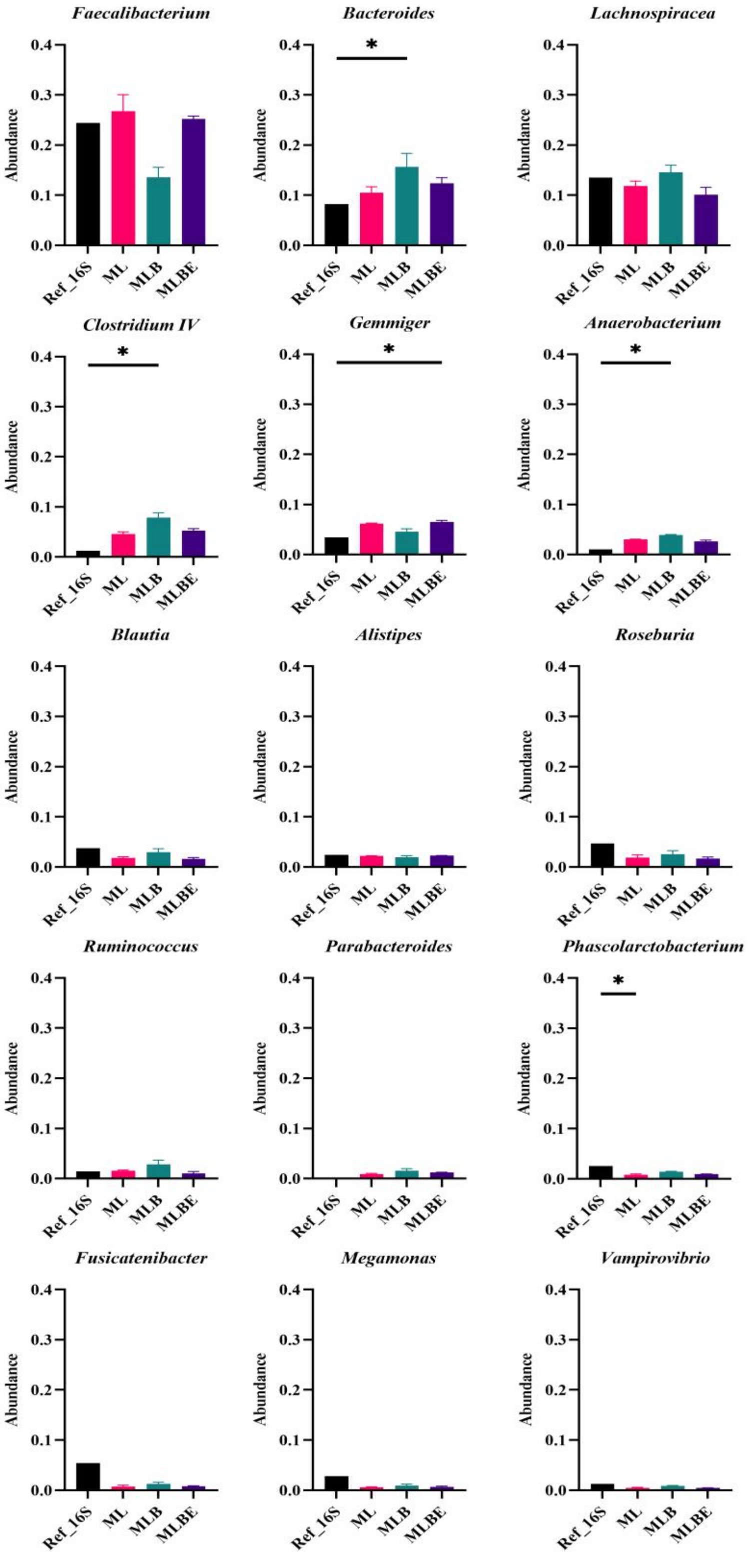

Figure S7. Relative abundance of Top 15 bacterial taxa at the species level. Kruskal-Wallis statistic test; asterisks indicate adjusted *p*-value < 0.05 in Dunn's multiple comparisons posthoc test. Error bars are indicated on top of the boxes.

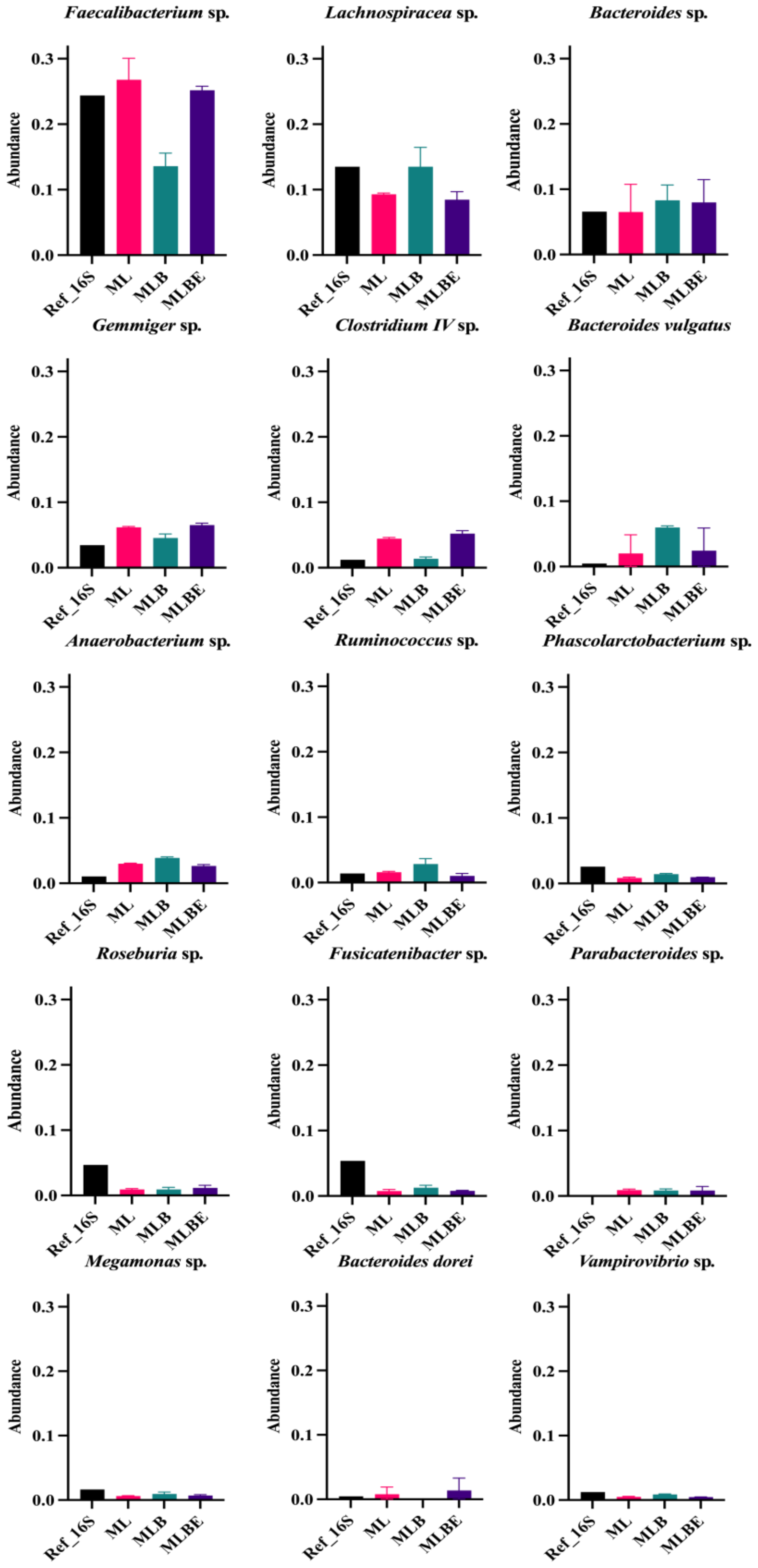

Figure S8. Relative abundance of all fungal taxa identified at the genus level. Kruskal-Wallis statistic test; asterisks indicate adjusted p-value < 0.05 in Dunn's multiple comparisons posthoc test. Error bars are indicated on top of the boxes.

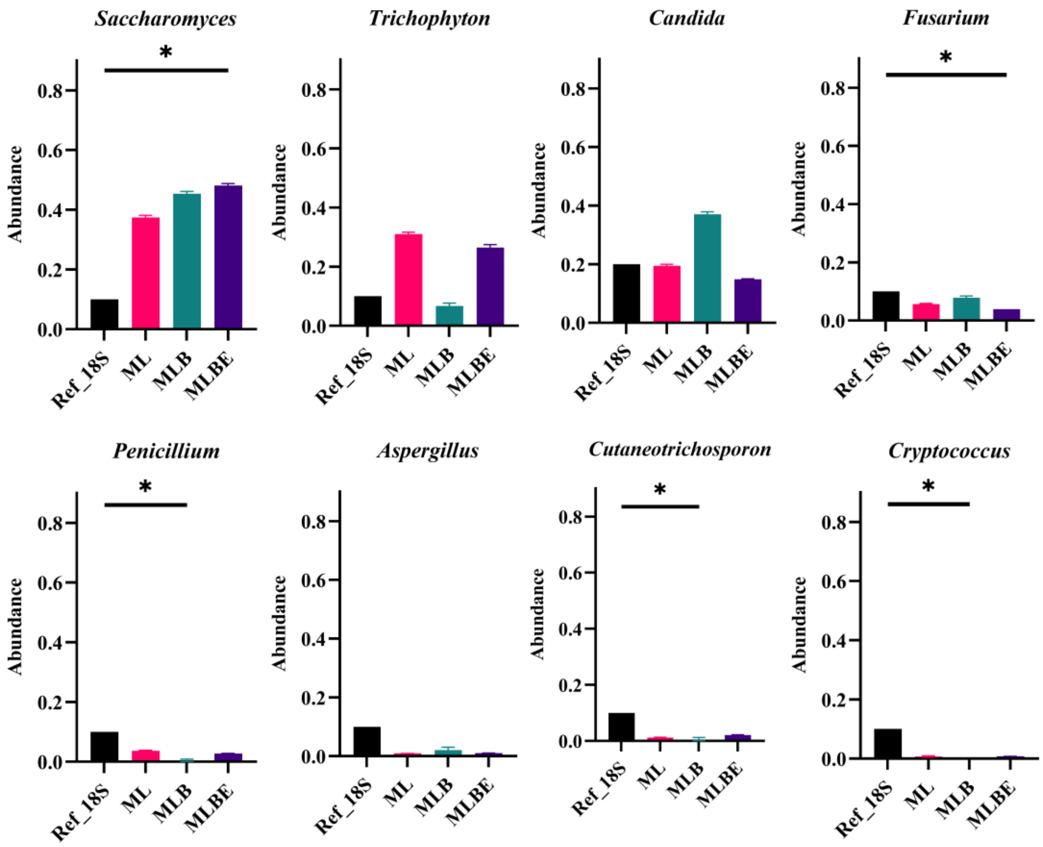

Figure S9. Relative abundance of all fungal taxa identified at the species level. Kruskal-Wallis test; asterisks indicate adjusted p-value < 0.05 in Dunn's multiple comparisons posthoc test. Error bars are indicated on top of the boxes.

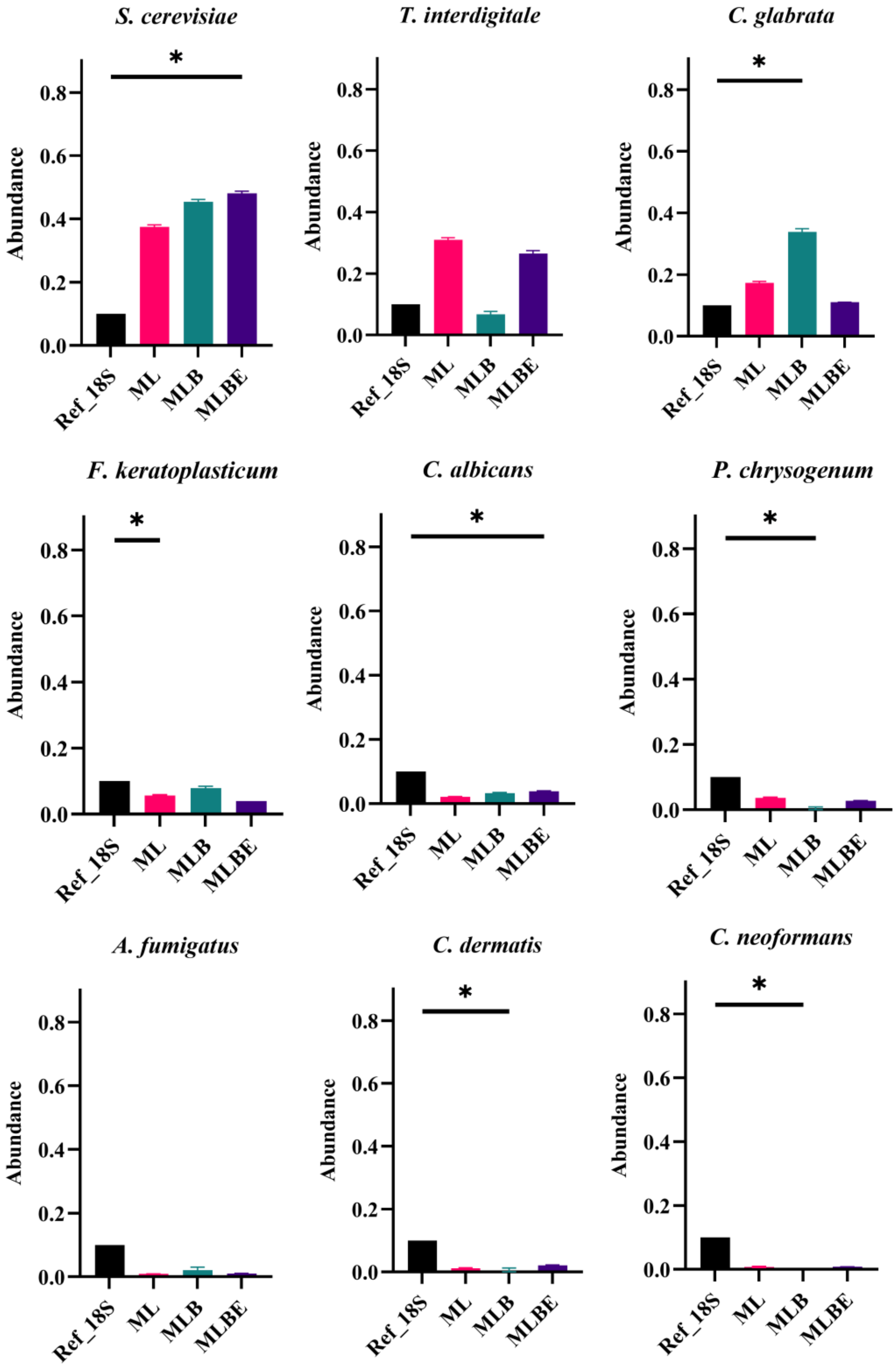

Supplement: Uncited Supplementary Material 1. [file acmi-6-00754-s001.pdf]
